# SAIL study of stroke, systemic embolism and bleeding outcomes with warfarin anticoagulation in non-valvular atrial fibrillation (S^4^-BOW-AF)

**DOI:** 10.1093/ehjopen/oead037

**Published:** 2023-04-13

**Authors:** Daniel E Harris, Fatemeh Torabi, Daniel Mallory, Ashley Akbari, Daniel Thayer, Ting Wang, Sarah Grundy, Mike Gravenor, Raza Alikhan, Steven Lister, Julian Halcox

**Affiliations:** Population Data Science, Swansea University, Singleton Park, Swansea, SA28PP, UK; Tritech Institute, Hywel Dda University Health Board, Unit 2 Dura Park, Bynea, SA14 9TD, UK; Population Data Science, Swansea University, Singleton Park, Swansea, SA28PP, UK; Population Data Science, Swansea University, Singleton Park, Swansea, SA28PP, UK; Population Data Science, Swansea University, Singleton Park, Swansea, SA28PP, UK; Population Data Science, Swansea University, Singleton Park, Swansea, SA28PP, UK; Population Data Science, Swansea University, Singleton Park, Swansea, SA28PP, UK; Medical Department, Bristol-Myers Squibb ltd, ARC Uxbridge, Sanderson Road, Denham, UB8 1DH, UK; Population Data Science, Swansea University, Singleton Park, Swansea, SA28PP, UK; Thrombosis Centre, University Hospital of Wales, Heath Park, Cardiff, CF14 4XW, UK; Department of Health Economics, Bristol-Myers Squibb ltd, ARC Uxbridge, Sanderson Road, Denham, UB8 1DH, UK; Population Data Science, Swansea University, Singleton Park, Swansea, SA28PP, UK; Cardiology Department, Swansea Bay University Health Board, Sketty Lane, Swansea, SA28QA, UK

**Keywords:** Atrial fibrillation, Warfarin, INR control, Stroke, Bleeding, Pharmacotherapy

## Abstract

**Aims:**

In patients with non-valvular atrial fibrillation (NVAF) prescribed warfarin, the association between guideline defined international normalised ratio (INR) control and adverse outcomes in unknown. We aimed to (i) determine stroke and systemic embolism (SSE) and bleeding events in NVAF patients prescribed warfarin; and (ii) estimate the increased risk of these adverse events associated with poor INR control in this population.

**Methods and results:**

Individual-level population-scale linked patient data were used to investigate the association between INR control and both SSE and bleeding events using (i) the National Institute for Health and Care Excellence (NICE) criteria of poor INR control [time in therapeutic range (TTR) <65%, two INRs <1.5 or two INRs >5 in a 6-month period or any INR >8]. A total of 35 891 patients were included for SSE and 35 035 for bleeding outcome analyses. Mean CHA_2_DS_2_-VASc score was 3.5 (SD = 1.7), and the mean follow up was 4.3 years for both analyses. Mean TTR was 71.9%, with 34% of time spent in poor INR control according to NICE criteria.

SSE and bleeding event rates (per 100 patient years) were 1.01 (95%CI 0.95–1.08) and 3.4 (95%CI 3.3–3.5), respectively, during adequate INR control, rising to 1.82 (95%CI 1.70–1.94) and 4.8 (95% CI 4.6–5.0) during poor INR control.

Poor INR control was independently associated with increased risk of both SSE [HR = 1.69 (95%CI = 1.54–1.86), *P* < 0.001] and bleeding [HR = 1.40 (95%CI 1.33–1.48), *P* < 0.001] in Cox-multivariable models.

**Conclusion:**

Guideline-defined poor INR control is associated with significantly higher SSE and bleeding event rates, independent of recognised risk factors for stroke or bleeding.

## Graphical Abstract

**Graphical abstract:**
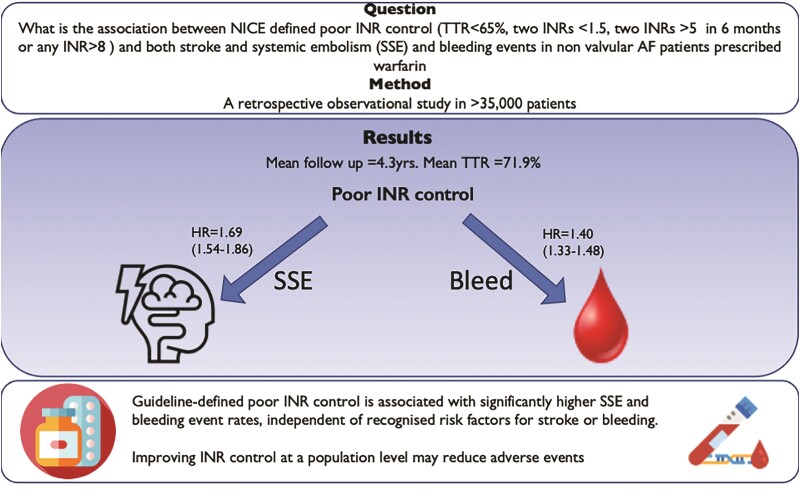

## Introduction

Historically, vitamin K antagonists (VKA) therapy has been the anticoagulant of choice to reduce the risk of stroke in patients with non-valvular atrial fibrillation (NVAF).^1^ However, successful VKA therapy has important practical limitations, including regular monitoring of patients’ international normalised ratio (INR) due to variability of control.^2,3^ The target INR range is 2–3 (unless otherwise indicated)^4–8^ with net clinical benefit closely related to the proportion of time that INRs remain in this range [time in therapeutic range (TTR)].^9–11^ Subtherapeutic INRs are associated with increased risk of stroke and systemic embolism (SSE), while supratherapeutic INRs increase bleeding risk.^9,12,13^

Guidelines stress the importance of assessing INR control, achieving acceptable TTR, and re-evaluating therapy if adequate control cannot be achieved. The UK National Institute for Health and Care Excellence (NICE) defines poor anticoagulation as any of the following: (i) TTR <65%; (ii) two INR values >5 or one >8 within the past 6 months; and (iii) two INR values <1.5 within the past 6 months.^6^ The European Society of Cardiology (ESC)^14^ and United States (US)^5^ guidelines recommend a TTR of ≥70%. Major clinical guidelines now recommend that anticoagulation with direct oral anticoagulants (DOACs) should also be considered where appropriate.^5,6,14^

We have previously demonstrated in a large population that a considerable proportion of patients exhibited suboptimal INR control according to NICE and ESC guideline criteria.^15^ However, the magnitude of the impact on major adverse outcomes of suboptimal control according to NICE clinical guideline criteria (which also include ‘High/Low’ criteria for effective control as well as TTR) has not been demonstrated. We were also interested to see if outcomes differed in those with evidence of poor control according to NICE criteria to those with inadequate control according to ESC and US criteria, which only consider the TTR.

We aimed to (i) determine SSE and bleeding event rates in patients prescribed warfarin for NVAF, and (ii) estimate the incremental risk of these adverse events associated with poor INR control (defined using NICE and ESC/US guideline criteria), accounting for patient clinical and demographic characteristics.

## Methods

A population-scale retrospective observational cohort study was conducted using individual-level linked anonymised routine electronic health record data sources for patients prescribed warfarin for NVAF in Wales, United Kingdom, between January 2006 and December 2017 using the Secure Anonymised Information Linkage (SAIL) Databank.^16–18^

### Cohort selection

Patients eligible for the study had a diagnosis of AF/atrial flutter recorded in their primary care record at any point before or during the study period (2006–2017) and were ≥18 years old at time of diagnosis. Patients were excluded if they had valvular AF (AF in the presence of mitral stenosis, rheumatic mitral valve disease, prior mitral valve surgery, metallic prosthetic heart valve) or venous thromboembolism (VTE) including deep vein thrombosis (DVT) and pulmonary embolism (PE) before, or within 6 months of inclusion, or were pregnant during the study period. Patients who were subsequently diagnosed with a VTE or valvular AF after 6 months from entry into the study were censored at the point of new diagnosis.

The cohort was restricted to those prescribed warfarin and with ≥6 months of recurrent INR tests recorded in their primary care record during the study period (excluding the first 6 weeks after commencing treatment, whilst the warfarin dose is typically being tailored to patient requirements).

### Temporal calculation of INR control

**Figure 1:**
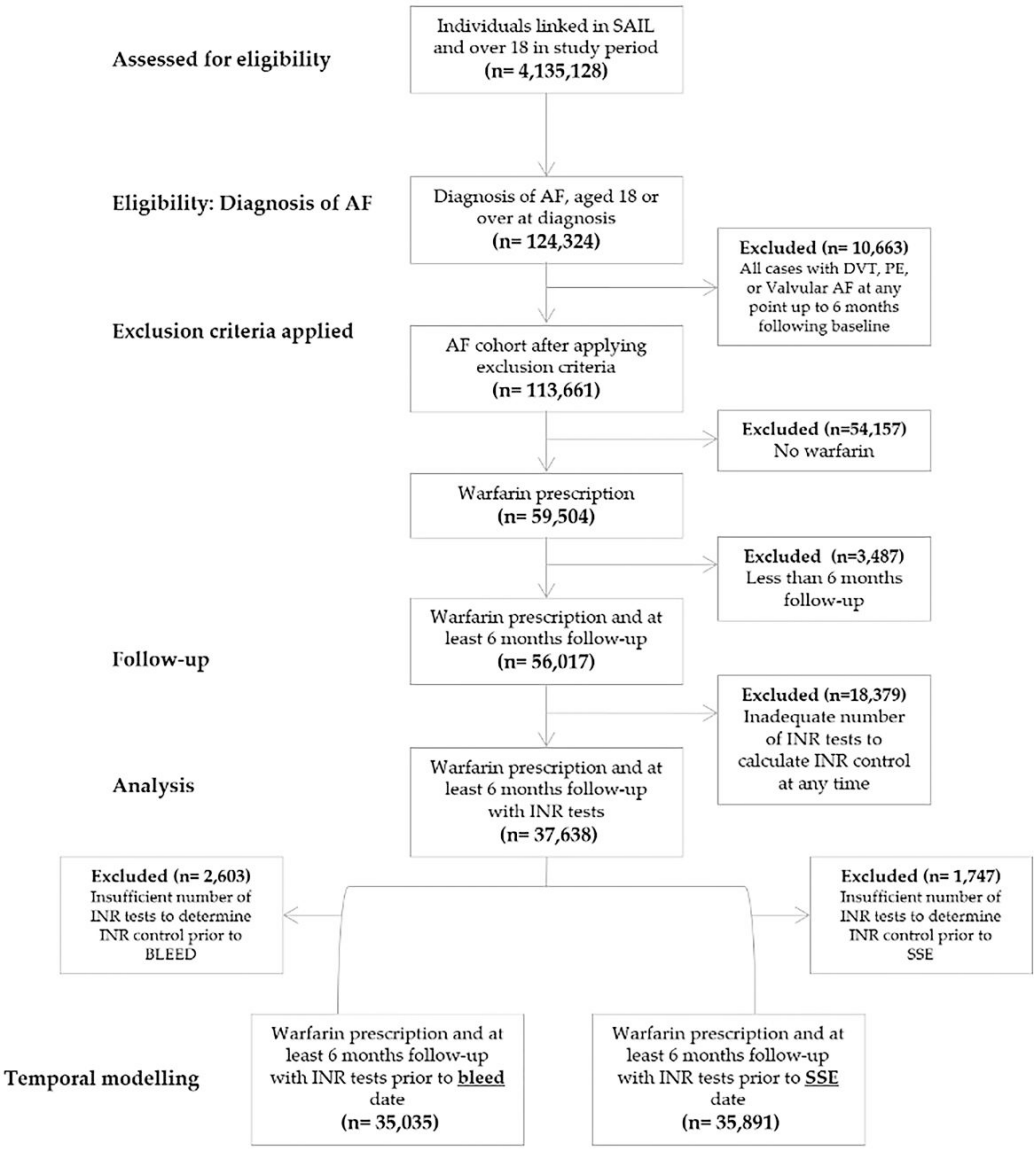
Inclusion criteria for study cohort.

Individual TTR was calculated at each INR result within 6-month rolling windows using the modified Rosendaal method.^19^ We also stipulated that there be: (i) at least four INR readings within each 6-month period; (ii) no gap >12 weeks between consecutive INR readings, and (iii) a gap of at least 3 months between the first and last INR reading within each 6-month window (Supplementary material Temporal INR control, *Figure 1 & 2A–D*); and (iv) at least one warfarin prescription in any 12-week window.^15^

Based on these criteria, an algorithm was developed to allow the temporal calculation of INR control, and assign patients to ‘unknown,’ ‘adequate,’ or ‘poor’ INR control status at each INR reading. Two established guideline criteria for poor INR control were assessed: Firstly, NICE criteria for poor INR control, one (or more) of the following (i) TTR <65%; (ii) two INR values higher than 5 within the prior 6 months; or a single INR value higher than 8; and (iii) two INR values less than 1.5 within the past 6 months. Secondly, ESC/US criteria for poor INR control, defined as periods of TTR <70% (secondary analysis).

Patients could move between adequate, poor, and unknown control status; assignment of patients to unknown status would result in temporary exclusion from analysis for any period during which there were insufficient INR results or no warfarin prescriptions available. Patients could re-enter the analysis when another six-month window became available with sufficient INR results and warfarin prescriptions for evaluation.

An index date was assigned to each patient when all inclusion criteria were first met. The number of days a patient was in adequate and/or poor control was calculated to the end of 2017. Patients were censored at death or when an adverse event occurred (see below), including those adverse events occurring outside of periods of INR calculation or when lost to follow-up (end of primary care record).

### Adverse events

Adverse events comprised (i) SSE and (ii) bleeding events (categorised as gastrointestinal, urinary, respiratory, intracranial, gynaecological, ocular, or miscellaneous bleeds in other organ systems) recorded in either the primary or secondary care records (see supplementary material online, *Tables S1A-D* for diagnostic codes).

Two cohorts were created to analyse the association between poor INR and SSE events and bleeding events. Adverse events occurring during periods of INR calculation and within 84 days of the last warfarin prescription were included, and patients were classified as having poor or adequate INR control based on the preceding 6 months of INR data. Finally, events occurring during periods where it was not possible to calculate INR control due to insufficient INR readings were excluded from our analysis.

### Medical history, demographic information, and prescriptions

Demographic and clinical data (reflecting standard stroke and bleeding risk classification,^20,21^ and comorbidities of major organ systems) prior to the index date for each patient were identified. Age and deprivation quintile^22^ were assigned at the index date. Heart failure, hypertension, vascular disease [defined as prior myocardial infarction (MI) or peripheral vascular disease (PVD) including peripheral artery disease and aortic plaque], prior stroke [including transient ischaemic attack (TIA)], diabetes, sex, and age were used to calculate the individual CHA_2_DS_2_-VASc score at the index date.^20^

### Statistical methods

Baseline characteristics of patients experiencing SSE or bleeding events during periods of INR calculation were compared to those without respective events using chi-squared and ANOVA tests as appropriate.

SSE and bleeding event rates were calculated during periods of adequate or poor INR control using each guideline’s thresholds for INR control. Relationships between INR control, SSE and bleeding events were then calculated using NICE criteria. Since our data allowed estimation of times during which patients move between poor and adequate INR control status, we considered INR control as a time-dependent variable and estimated hazard ratios (HRs) representing the risk at any specific time point. Initial multivariable-models utilized Cox-regression to estimate the risk of SSE or bleed according to INR control status, adjusting for the baseline individual CHA_2_DS_2_-VASc score and deprivation quintile. Secondary analyses were then conducted using ESC/US guideline criteria for poor control, using the same statistical approaches. Further Cox-regression models examined relationships between INR control (time-dependent) and SSE and bleeding outcomes, adjusting for relevant individual risk factors, including components of CHA_2_DS_2_-VASc score [including age, sex, and the presence or absence of following: heart failure, hypertension, age, diabetes mellitus, stroke (including TIA), sex, and vascular disease (defined as prior MI, PVD, or aortic plaque)]. Analyses were performed using IBM SPSS v26 and R version 3.5.3.

### Missing data

Comparisons were made between those included in the final cohorts for analysis and (i) those with NVAF prescribed warfarin but with inadequate or no INR test results for analysis, and (ii) those with insufficient INR tests recorded in the primary care dataset to classify INR control prior to either a SSE or bleed. Finally, within the final cohort, comparisons were made between those with and without deprivation quintile data available. Differences in these characteristics between groups were summarised using chi-squared tests for categorical variables and independent *t*-tests for continuous variables.

### Results

Over 4 million patient records were identified in the SAIL Databank during the study period; 124324 patients had a diagnosis of AF and aged over 18 at diagnosis of which 10 633 had a diagnosis of DVT, PE, or valvular prior to or within 6months of the study. A total of 37638 were prescribed warfarin with ≥6 months INR data (*Figure 1*) (see supplementary material online, *Table S2*).

We excluded 1747 from the final analyses who had an SSE and 2603 that bled during the study period but had inadequate number of INR results in the 6 months prior to the event to allow calculation of INR control. A total of 410 patients were censored during the study period due to receiving a diagnosis of valvular AF and a further 2024 patients were censored due to a DVT or PE.

A total of 35 891 patients had sufficient data to analyse associations between INR control and SSE, and 35 035 patients had sufficient data to be included in the bleeding analysis. Both cohorts had a mean follow up of 4.3 years, mean TTRs of 71.9%, and mean CHA_2_DS_2_-VASc score of 3.5 (SD = 1.7). The percentage of time spent with poor INR control using the NICE criteria was 34.0% and using ESC/US criteria was 40.9%, for both SSE and bleeding outcome analyses.

### SSE cohort

Over the study period, a total of 2802 SSE events occurred in 2422 patients. During periods where there were sufficient data to allow calculation of INR control, 1868 SSE events occurred in 1837 patients. Of those with SSE events, 1650 were strokes and 218 were non-stroke systemic emboli.

Patients experiencing SSE during the study period tended to be older, have higher CHA_2_DS_2_-VASc score, and higher prevalence of hypertension, prior ischemic stroke and PVD (*Table 1*) than those who did not. Females were less likely to suffer SSE than males.

**Table 1:** Cohort baseline characteristics of those with and without stroke & systemic embolism events

	SSE Cohort	Patients with SSE events	Patients without SSE events	*P* value^a^
	*n* (%)	*n* (%)	*n* (%)	
	*n* = 35 891	*n* = 1837	*n* = 34 054	
	*n* (%)	*n* (%)	*n* (%)	
Age				<0.001
18–64	5853 (16.3)	165 (9.0)	5688 (16.7)	
65–74	11 925 (33.2)	543 (29.6)	11 382 (33.4)	
75+	18 113 (50.5)	1129 (61.5)	16 984 (49.9)	
Female	15 328 (42.7)	877 (47.7)	14 451 (42.4)	<0.001
Deprivation index (quintile)*				0.12
1 (most deprived)	5839 (16.3)	325 (17.7)	5514 (16.2)	
2	6728 (18.7)	331 (18.0)	6397 (18.8)	
3	7690 (21.3)	360 (19.6)	7330 (21.5)	
4	6770 (18.9)	356 (19.4)	6423 (18.9)	
5 (least deprived)	7459 (20.7)	403 (21.9)	7056 (20.7)	
Deprivation index unknown	1405 (3.9)	62 (3.4)	1334 (3.9)	
CHA_2_DS_2_-VASc score				<0.001
0 and 1	4122 (11.5)	76 (4.1)	4046 (11.9)	
2	6272 (17.5)	203 (11.1)	6069 (17.8)	
3	8154 (22.7)	365 (19.9)	7789 (22.9)	
4	7901 (22.0)	418 (22.8)	7483 (22.0)	
5	5026 (14.0)	375 (20.4)	4651 (13.7)	
6	2914 (8.1)	254 (13.8)	2660 (7.8)	
≥7	1502 (4.2)	146 (7.9)	1356 (4.0)	
Heart failure	8475 (23.6)	417 (22.7)	8058 (23.7)	0.36
Hypertension	23 581 (65.7)	1344 (73.2)	22 237 (65.3)	<0.001
Diabetes	7666 (21.4)	426 (23.2)	7240 (21.3)	0.05
Ischemic stroke	7068 (19.7)	667 (36.3)	6401 (18.8)	<0.001
Thromboembolism	478 (1.3)	37 (2.0)	441 (1.3)	0.012
Ischemic heart disease	10 909 (30.4)	594 (32.3)	10 315 (30.3)	0.06
Peripheral Vascular Disease	2186 (6.1)	193 (10.5)	1993 (5.9)	<0.001
Liver disease	687 (1.9)	30 (1.6)	657 (1.9)	0.42
Chronic Kidney disease (stage 4+)	435 (1.2)	22 (1.2)	413 (1.2)	1
Excessive alcohol intake	938 (2.6)	61 (2.6)	877 (2.6)	0.71
Any prior bleeding	4919 (13.7)	333 (14.3)	4586 (13.7)	0.22

*Deprivation index was calculated using the Welsh Index of Multiple Deprivation 2011 quintiles.

a *P* value calculated using a pair-wise comparison of subgroups with and without SSE during follow up.

### Estimates of the effect of INR control on risk of SSE

In univariable Cox analyses, the HR for SSE associated with poor INR control according to NICE criteria was 1.84 [(95%CI 1.68–2.02), *P* < 0.001]; using the ESC/US criteria for poor control the HR for SSER was1.84 (95%CI 1.68–2.02).

In the first multivariable model, poor INR according to NICE criteria was independently associated with an increased risk of SSE after adjustment for CHA_2_DS_2_-VASc score and deprivation quintile (*Table 2*). CHA_2_DS_2_-VASc score was also independently associated with SSE, after mutual adjustment, while there was no significant association with deprivation level.

**Table 2:** Multivariable cox-regression model for hazard of stroke & systemic embolism events determined by poor INR control according to NICE criteria

	Stroke & systemic embolism
	HR (95%CI), *P* value
Poor INR control	1.69 (1.54–1.86), <0.001
CHA_2_DS_2_-VASc score	
0 &1	Reference
2	1.96 (1.50–2.56), <0.001
3	2.94 (2.29–3.77), <0.001
4	3.76 (2.94–4.83), <0.001
5	5.47 (4.26–7.04), <0.001
6	6.79 (5.22–8.13), <0.001
7	9.08 (6.85–12.05), <0.001
Deprivation index (quintiles)*	
1 (most deprived)	Reference
2	0.88 (0.76–1.04), 0.13
3	0.89 (0.76–1.03), 0.12
4	0.99 (0.85–1.15), 0.90
5 (least deprived)	0.92 (0.80–1.07), 0.28

* Deprivation index was calculated using the Welsh Index of Multiple Deprivation 2011 quintiles.

Results are adjusted for CHA_2_DS_2_-VASc score and deprivation quintiles. Any changes in INR control status for individuals over time were included in the model as a time dependent variable.

Poor INR control according to NICE criteria was again associated with SSE independently of other individual risk factors in the second set of models (*Table 3*). Increasing age was also associated with SSE, as was diabetes, prior ischaemic stroke and prior bleeding events, hypertension, PVD, and female sex, after mutual adjustment.

**Table 3:** Multivariable cox-regression model for hazard of stroke and systemic embolism determined by poor INR control according to NICE criteria

Predictor	SSE
	HR (95%CI), *P* value
Poor INR control	1.73 (1.57–1.89), <0.001
Female	1.11 (1.01–1.22), 0.03
Age	
<65	Reference
65–74	1.70 (1.43–2.03), <0.001
≥75	2.80 (2.36–3.32), <0.001
Excessive alcohol consumption	1.32 (0.97–1.79), 0.07
Heart failure	1.03 (0.92–1.16), 0.56
Hypertension	1.27 (1.14–1.41), <0.01
Diabetes	1.19 (1.07–1.34), 0.001
Ischemic stroke	2.17 (1.97–2.39), <0.001
Thromboembolism	1.24 (0.89–1.74), 0.19
Ischemic heart disease	0.99 (0.90–1.10), 0.94
Peripheral vascular disease	1.79 (1.53–2.09), <0.001
Liver disease	0.99 (0.69–1.44), 0.99
Chronic kidney disease stage 4+	1.19 (0.78–1.83), 0.40
Prior bleeding events	1.15 (1.01–1.31), 0.03

Results are adjusted for individual components of CHA_2_DS_2_-VASc score, plus baseline characteristics. Any changes in INR control status for individuals over time were included in the model as a time dependent variable.

Very similar relationships were seen between poor INR control according to ESC/US and SSE events in the multivariable models (see supplementary material online, *Tables S3 & 4*).

### SSE event rate

The SSE event rate (per 100 patient years) was 1.3 (95%CI 1.2–1.4) in the overall population; with a rate of 1.0 (95%CI 0.9–1.1) during periods of adequate INR control rising to 1.8 (95%CI 1.7–1.9) during periods of poor INR control according to NICE criteria. Very similar event rates were seen in those meeting ESC/US criteria for poor INR control (see supplementary material online, *Table S5*).

### Bleeding cohort

Across the entire study period, a total 7220 bleeds occurred in 6304 patients. During periods where there were sufficient readings to allow calculation of INR control, 5766 bleeds occurred in 5039 patients.

Patients who had bled tended to be older, have higher CHA_2_DS_2_-VASc score, had a higher prevalence of hypertension, prior ischemic stroke, ischemic heart disease and a history of prior bleeding events. (*Table 4*). Females were less likely to bleed, and deprivation index was also associated with bleeding risk in univariable analysis.

**Table 4:** Cohort characteristics of those with and without bleeding events

	Cohort	Patients with bleeding events	Patients without bleeding events	*P* value^a^
	*n* (%)	*n* (%)	*n* (%)	
	*n* = 35 035	*n* = 5039	*n* = 29 996	
Age				0.001
18–64	5679 (16.2)	725 (14.4)	4954 (16.5)	
65–74	11 610 (33.1)	1703 (33.8)	9907 (33.0)	
75+	17 746 (50.7)	2611 (51.8)	15 135 (50.5)	
Female	15 041 (42.9)	2011 (39.9)	13 030 (43.5)	<0.001
Deprivation index (quintile)				0.009
1 (most deprived)	5732 (16.3)	832 (16.5)	4900 (16.3)	
2	6573 (18.8)	945 (18.8)	5628 (18.8)	
3	7450 (21.3)	1027 (20.4)	6423 (21.4)	
4	6611 (18.9)	915 (18.2)	5696 (19.0)	
5 (least deprived)	7296 (20.8)	1143 (22.6)	6153 (20.5)	
Deprivation index unknown	1373 (3.9)	177 (3.5)	1196 (3.9)	
CHA_2_DS_2_-VASc score				0.001
0 and 1	3971 (11.3)	485 (9.6)	3486 (11.6)	
2	6018 (17.2)	815 (16.2)	5203 (17.3)	
3	7859 (22.4)	1170 (23.2)	6689 (22.3)	
4	7692 (22.0)	1128 (22.4)	6564 (21.9)	
5	4999 (14.3)	764 (15.2)	4235 (14.1)	
6	2962 (8.5)	470 (8.9)	2492 (8.4)	
≥7	1534 (4.4)	240 (4.5)	1294 (4.4)	
Heart failure	8283 (23.6)	1223 (24.3)	7060 (23.5)	0.26
Hypertension	23 049 (65.8)	3558 (67.1)	19 492 (65.6)	0.03
Diabetes	7476 (21.3)	1182 (22.3)	6294 (21.2)	0.07
Ischemic stroke	7291 (20.8)	1163 (23.1)	6128 (20.4)	<0.001
Thromboembolism	486 (1.4)	67 (1.3)	419 (1.4)	0.44
Ischemic heart disease	10 608 (30.3)	1721 (34.2)	8887 (29.6)	<0.001
Peripheral Vascular Disease	2139 (6.1)	326 (6.5)	1813 (6.0)	0.24
Liver disease	670 (1.9)	97 (1.9)	573 (1.9)	0.94
Chronic Kidney disease (stage 4+)	417 (1.2)	65 (1.3)	352 (1.2)	0.48
Excessive alcohol intake	935 (2.7)	117 (2.3)	818 (2.7)	0.10
Any prior bleeding	4657 (13.3)	870 (17.3)	3787 (12.6)	<0.001

*Deprivation index was calculated using the Welsh Index of Multiple Deprivation 2011 quintiles.

a *P* value calculated using a pair-wise comparison of subgroups with and without SSE during follow up.

**Table 5:** Multivariable cox-regression model for hazard of bleeding events determined by poor INR control according to NICE criteria

	Bleed
	HR (95%CI), *P* value
Poor INR control	1.40 (1.33–1.48), <0.001
CHA_2_DS_2_-VASc score	
0 &1	Reference
2	1.22 (1.09–1.37), <0.001
3	1.45 (1.30–1.61), <0.001
4	1.54 (1.38–1.72), <0.001
5	1.67 (1.48–1.87), <0.001
6	1.68 (1.47–1.92), <0.001
7	1.99 (1.70–2.34), <0.001
Deprivation index (quintiles)^a^	
1 (most deprived)	Reference
2	0.99 (0.91–1.10), 0.99
3	0.96 (0.87–1.05), 0.36
4	0.96 (0.88–1.06), 0.45
5 (least deprived)	1.00 (0.92–1.10), 0.97

a Deprivation index was calculated using the Welsh Index of Multiple Deprivation 2011 quintiles.

Results are adjusted for CHA_2_DS_2_-VASc score and deprivation quintiles. Any changes in INR control status for individuals over time were included in the model as a time dependent variable.

**Table 6:** Multivariable cox-regression model for hazard of bleeding events determined by poor INR control according to NICE criteria

Predictor	Bleed
	HR (95%CI), *P* value
Poor INR control	1.38 (1.31–1.46), <0.001
Female	0.85 (0.80–0.90), <0.001
Age	
<65	Reference
65–74	1.26 (1.16–1.38), <0.001
≥75	1.62 (1.48–1.77), <0.001
Heart failure	1.08 (1.01–1.15), 0.03
Hypertension	1.05 (0.99–1.12), 0.07
Diabetes	1.12 (1.05–1.20), <0.001
Ischemic stroke	1.10 (1.03–1.17), 0.005
Thromboembolism	0.93 (0.73–1.20), 0.59
Ischemic heart disease	1.16 (1.10–1.24), <0.001
Peripheral Vascular Disease	1.11 (0.98–1.24), 0.09
Liver disease	1.15 (0.94–1.41), 0.19
Chronic Kidney disease (stage 4+)	1.45 (1.13–1.85), 0.003
Excessive alcohol consumption	0.96 (0.79–1.17), 0.71
Prior bleeding events	1.55 (1.44–1.67), <0.001

Results are adjusted for individual components of CHA_2_DS_2_-VASc score, plus baseline characteristics. Any changes in INR control status for individuals over time were included in the model as a time dependent variable.

### Estimates of the effect of INR control on risk of bleed

In univariable Cox analyses, the HR for bleeding associated with poor control according to NICE criteria was 1.43 [(95% CI 1.35–1.51), *P* < 0.001]. Using the ESC/US criteria of poor INR control as a univariable the HR for bleeding was 1.45 (95%CI 1.38–1.54).

In the first multivariable models, poor INR (according to NICE criteria) was associated with both an increased risk of bleeding (*Table 5*) after adjustment for CHA_2_DS_2_-VASc score, and deprivation quintile. CHA_2_DS_2_-VASc score was also independently associated with bleeding events, after mutual adjustment but deprivation quintile was no longer associated.

Poor INR control according to NICE criteria was again associated with bleeding independently of other risk factors in the second multivariable model (*Table 6*). After mutual adjustment, increasing age was also associated bleeding events, as was diabetes, prior ischaemic stroke, and prior bleeding events, ischaemic heart disease, heart failure, and chronic kidney disease (stage 4+) was associated with bleeding events. Female sex was associated with fewer bleeds.

Very similar relationships were seen between poor INR control according to ESC/US and bleeds in the multivariable models (see supplementary material online, *Tables S3 & S4*).

### Bleeding event rate

The bleeding event rate was 3.9 (95%CI 3.8–4.0) in the overall population; 3.4 (95% CI, 3.3–3.5) during periods of adequate INR control and rising to 4.8 (95% CI 4.6–5.0) during periods of poor INR control according to the NICE criteria. Very similar bleeding event rates were seen in those meeting ESC/US criteria for poor INR control (see supplementary material online, *Table S6*).

### Discussion

This is the first real-world study from a national cohort that has assessed the association between INR control according to major clinical guideline criteria and both SSE and bleeding events from the clinical records of individual patients prescribed warfarin for NVAF. Evidence of poor INR control was present in over one-third of the evaluated monitoring period at a population level and independently associated with a significant increased risk of both SSE and bleeding events.

Despite a greater proportion of time spent in ‘poor’ INR control with the ESC/US (40.9%) compared to the NICE criteria (34.0%), the relationships between SSE or bleeding event rates and poor INR control were very similar in magnitude with both guidelines.

The overall SSE and bleed rate in this study was 1.3 and 3.9 per 100 patient years, respectively. This was similar to rates recently reported in another real-world study of INR control in patients with AF,^12^ as well as those reported in randomised controlled studies where VKA therapy was included as a comparator against the DOACs (SSE range 1.50–2.2% and bleed range 3.1–3.4% in VKA arms).^23–26^

While the mean TTR in this study was 72% compared to 55–64% in randomized controlled trials (RCTs), the lower bleeding event rate observed in the RCTs may be explained by differences in definition of bleeding events, selective patient enrolment and enhanced observation of patients compared to real-world studies.^23–26^ Differences in methods of calculating TTR, and the absence of reporting INR control assessed by very low or very high individual INRs, limits the comparisons that can be made between studies, and health care systems. Lastly, the comparison in bleeding event rate between studies is further limited by the lack of consistent reporting of bleeding events and severity.

This study evaluated the impact of multiple clinical and demographic factors as well as temporal INR control according to multiple criteria in one of the largest real-world studies of INR control in patients with NVAF. The use of a population-scale, individual-level rich linked anonymised data sources is a particular strength. The linked primary and secondary care data held by SAIL enable the investigation of a very large cohort of individuals longitudinally over a period of years and across multiple data sources, giving a much more complete picture of patient treatment, health, and clinical characteristics in a diverse and representative population than in previous studies.

Increasing stroke risk, assessed by the CHA_2_DS_2_-VASc score was, as expected, associated with an increased risk of both SSE and bleeding, as were many individual characteristics commonly associated with stroke or bleeding. Increasing age (≥75 years) was associated with the highest risk of SSE and bleeding. Prior bleeding events were also strongly associated with further bleeding events and prior ischaemic stroke was associated with SSE, as demonstrated in previous studies.^27–29^

We found that females had a lower likelihood of bleeds, but a slightly higher likelihood of SSE independently on INR control, in keeping with previous studies.^29,30^ Notably, INR control was worse in females than males, as we demonstrated previously in this cohort.^15^ It is therefore uncertain whether the sex imbalance in observed SSE outcomes could be diminished by improving INR control in women.

Surprisingly, we observed no independent association between deprivation and INR control, SSE or bleeding in this study. The data for this study were obtained from routine data sources following patient interactions within the Welsh National Health Service, where prescriptions and INR monitoring are free to patients at the point of delivery, potentially mitigating important financial access barriers to healthcare, as seen in more economically-disadvantaged individuals or populations. This should be an important consideration when comparing the findings of our study to other healthcare systems.

Deprivation index data were missing in ∼1.9% of patients and were therefore excluded from the first multivariable models (see supplementary material online, *Table S7A & B*). These patients had slightly lower prevalence of hypertension and a slightly higher prevalence of heart failure, but otherwise had very similar characteristics to the overall cohort. Importantly, all major comorbidities were well represented in the multivariable models and the inclusion of this small group would not be expected to have materially influenced the strong associations between poor INR control and adverse outcomes.

We excluded patients who also had a history of valvular AF and/or VTE as they may have had ‘individualised’ INR targets, which may not have been identifiable in the SAIL Databank and may potentially have biased the study towards a greater number of patients with ‘poor INR control’. Furthermore, our clinical experience suggests that these more complex patients are more often managed via specialist secondary care haematology-led anticoagulation services and their INR results may not have been available for analysis in this study.

We only evaluated patients prescribed warfarin during periods where their INR results were documented in the primary care record in this study. Patients monitored in hospital clinics and those who were undertaking home INR testing (albeit rarely) are unlikely to have their individual INR data entered consistently in their primary care record. It was also not possible to identify periods of temporary discontinuation of warfarin for patients undergoing surgery which if recorded in the primary care record may have resulted in periods of INR control recorded as out of range.

We identified a number of patients prescribed warfarin, with at least 6-month follow up time but who had inadequate number of INR results during the study period who were therefore excluded from the analyses. A further group were excluded who had an had inadequate number of INR results in the 6 months prior to the event to allow calculation of INR control (see supplementary material online, *Table S8A & B*). It cannot be determined why these patients had insufficient INR readings recorded in the respective period. However, these patients had a significantly higher prevalence of most SSE and bleeding risk factors, and it is likely that most of these patients were managed in secondary care, although it is also possible that they may not have been receiving warfarin nor having regular INR checks.

In this study, we identified the individual components of the CHA_2_DS_2_-VASc score as well as risk factors associated with SSE or bleeding at the index date of admission into the study, including at the time of diagnosis of AF for incident patients. It was beyond the scope of this study to recalculate the CHA_2_DS_2_-VASc score or identify new risk factors/comorbidities dynamically throughout the follow up period. It is unknown whether this may have added some incremental benefit or improved the accuracy in the associations between these variables and bleeding events. Regardless, the results in this large real-world population study are compelling; poor INR control and increasing stroke risk are independent markers of increased risk of both SSE and bleeding events.

The HASBLED score was not calculated in this study for several reasons; poor INR control (a component of the HASBLED score) was measured independently; pathology results, alcohol and illicit drug use are less robustly documented in the WLGP datasets, and aspirin and non-steroidal anti-inflammatory are frequently purchased without a prescription in the UK. Finally, the HASBLED score, unlike CHA_2_DS_2_-VASc, is at least partially modifiable, likely to change dynamically throughout the study period and does not provide significantly greater discrimination of bleeding risk than CHA_2_DS_2_-VASc at a population level.^31^

The temporal calculation of INR control allowed us to assign patients to adequate or poor INR control at each INR result based on the previous 6 months of INR data; periods where there were insufficient INR results were excluded from INR calculation but allowed patients to re-enter the analysis when there were sufficient INR results to recalculate INR control. While this conservative approach had the potential to exclude periods of INR calculation in patients who had planned extended periods between INR tests, fewer than 1.4% of INR tests had an interval of greater than 84 days. This approach provided greater surety that only periods of warfarin administration and monitoring were included in the calculation of INR control. Furthermore, the recalculation of INR control and assignment to adequate or poor INR control at each INR test allowed us to test the association between adverse events and INR control in the directly preceding period. Our approach provides a methodological improvement over previous studies that have reported the association between bleeding events and mean TTR, which may have been calculated over a period of years prior to an event.^9^

We found similar event rates in those with evidence of inadequate control by ESC criteria as those with inadequate control by NICE criteria at the population level. Whilst one could argue that the ESC guidelines could be adopted by the UK due to their greater simplicity, where clinical computer software can reliably identify those with ‘high’ or ‘low’ INR levels, this could still help guide individual patient management approaches and we would not advocate to change the UK guidance. However, our findings suggest that where those data are not readily available, following the ESC guidelines is acceptable in UK practice.

This study was conducted prior to the COVID-19 pandemic when a greater proportion of patients were prescribed warfarin. Many healthcare providers have since moved patients to DOAC therapy which requires less intensive monitoring and patient contact. However, we are aware that across many healthcare systems large numbers of patients are still prescribed warfarin. The data in this study may provide valuable insight into selecting patients who are at the highest risk of bleeding with warfarin, who may require the greatest effort in improving INR and could be considered for alternative anticoagulation strategies where clinically appropriate.

### Conclusion

Periods of poor INR control, as well as increasing stroke risk and specific comorbidities for stroke and bleeding, were associated with a considerable increase in the risk of SSE and bleeding events in warfarin treated patients with NVAF. The potential to reduce these adverse events through improvement in INR control at a population level should be closely considered to help improve patient outcomes. For individuals, a detailed risk assessment, considering factors leading to poor INR control and comorbidities that increase SSE and bleeding risk, remains essential. However, it is clear that improved measures to optimise effectiveness of anticoagulation are likely to improve clinical outcomes.

## Supplementary Material

oead037_Supplementary_Data

## Data Availability

The data used in this study are available in the SAIL Databank at Swansea University, Swansea, UK, but as restrictions apply they are not publicly available. All proposals to use SAIL data are subject to review by an independent Information Governance Review Panel (IGRP). Before any data can be accessed, approval must be given by the IGRP. The IGRP gives careful consideration to each project to ensure proper and appropriate use of SAIL data. When access has been granted, it is gained through a privacy protecting safe haven and remote access system referred to as the SAIL Gateway. SAIL has established an application process to be followed by anyone who would like to access data via SAIL at https://www.saildatabank.com/application-process.

## Lead author biography

Dr Daniel E. Harris has over 20 years’ experience in clinical pharmacy practice. He has recently been appointed as a clinical academic at Hywel Dda University Health Board, Wales, and as an Honorary Associate Professor in Pharmacoepidemiology at Swansea University. His clinical and scientific interests are focused on cardiovascular risk prevention and management. He is currently delivering a research programme using retrospective population healthcare data to inform and test the delivery of optimal risk factor management of patients with or at high risk of cardiovascular disease.
