# Supplementary material for: SAIL study of stroke, systemic embolism and bleeding outcomes with warfarin anticoagulation in non-valvular atrial fibrillation (S4-BOW-AF)
Source: Eur Heart J Open. 2023 Apr 13;3(3):oead037. doi: 10.1093/ehjopen/oead037 (PMC10153743; doi:10.1093/ehjopen/oead037)
Supplement: oead037_Supplementary_Data [file oead037_supplementary_data.zip › Supplementary.pdf]

Supplementary information

Table of Contents

Data sources **2**

Temporal INR Control **3**

Supplementary Figure 1: TTR Temporal Window Identification Algorithm 3

Examples of Exclusion Criteria **5**

Supplementary Figure 2a: 5

Supplementary Figure 2b: 6

Supplementary Figure 2C: 6

Supplementary Figure 2D: 7

Supplementary Table 1a. Read codes for bleeding events for primary care data. 8

Supplementary Table 1a continued. Read codes for bleeding events for primary care data. 9

Supplementary Table 1a continued. Read codes for bleeding events for primary care data. 11

Supplementary Table 1b. *ICD-10* codes for bleeding events for secondary care data. 12

Supplementary Table 1b continued. *ICD-10* codes for bleeding events for secondary care data. 13

Supplementary Table 1b continued. ICD-10 codes for bleeding events for secondary care data. 14

Supplementary Table 1c. Read codes for SSE events for primary care data 15

Supplementary Table 1c continued. Read codes for SSE events for primary care data. 16

Supplementary Table 1d. *ICD-10* codes for SEE events for secondary care data 17

Supplementary Table 1d continued. *ICD-10* codes for SEE events for secondary care data 18

Table 2. Cohort baseline characteristics 19

Supplementary Table 3. Multivariable Cox-regression model for hazard of stroke and systemic embolism and bleeding events determined by poor INR control according to ESC/US criteria. 20

Supplementary Table 4. Multivariable Cox-regression model for hazard of stroke and systemic embolism and bleeding events determined by poor INR control ESC/US. 21

Supplementary Table 5. Stroke and systemic embolism event rate according to INR guideline criteria. 22

Supplementary Table 6. Bleeding event rate according to INR guideline criteria. 23
Supplementary Table 7a Comparisons between those with deprivation index present and missing from the final cohort included in the SSE analysis. 23
Supplementary Table 7b Comparisons between those with deprivation index present and missing from the final cohort included in the bleeding analysis. 24
Supplementary Table 8a. Cohort characteristics and comparisons to patients with inadequate number of INR readings to calculate INR control prior to a SSE and those with inadequate number of INR results to calculate INR control across any period during the study. 25
Supplementary Table 8b. Cohort characteristics and comparisons to patients with inadequate number of INR readings to calculate INR control prior to a bleed and those with inadequate number of INR results to calculate INR control across any period during the study. 27

Data sources

The following data sources held within SAIL were linked at individual-level: the Patient Episode Database for Wales (PEDW), which records hospital admission and discharge dates, diagnoses and operational procedures, demographic data, and date of death (where applicable) for the population of Wales; the Welsh Longitudinal General Practice (WLGP) data containing demographic, clinical, and prescribing data for approximately 80% of primary care practices across Wales; the Welsh Demographic Dataset (WDSD), which contains basic demographic information and history of individuals' residence in Wales, their registration history with General Practices (GP); and Lower-layer Super Output Area (LSOA) 2001 which is used to identify the Welsh Index of Multiple Deprivation (WIMD) 2011, an area-based deprivation measure. Further information on the data sources can be found here:

https://saildatabank.com/saildata/sail-datasets/

https://web.www.healthdatagateway.org/search?search=sail&tab=Datasets

Temporal INR Control

Our algorithm identifies valid temporal windows to identify sufficient readings to allow us to perform TTR calculations. As illustrated in supplementary Figure 1, temporal windows for each patient starts from 42-days after the date which marks the earliest point at which a patient has both had an INR test and received a prescription for warfarin (this is defined as the study start date for the patient). The initial 42-day period is not analysed because the patient’s INR is stabilising for this initial period. The existence of records in 6-monthly rolling windows will then be checked and we require at least 4 INR readings in each window to then contribute toward calculation of TTR for the patient. The final result of this algorithm is then used for further analysis to evaluate the bleeding outcomes while taking into the account any changes which might happened in the level of controls during the study period.

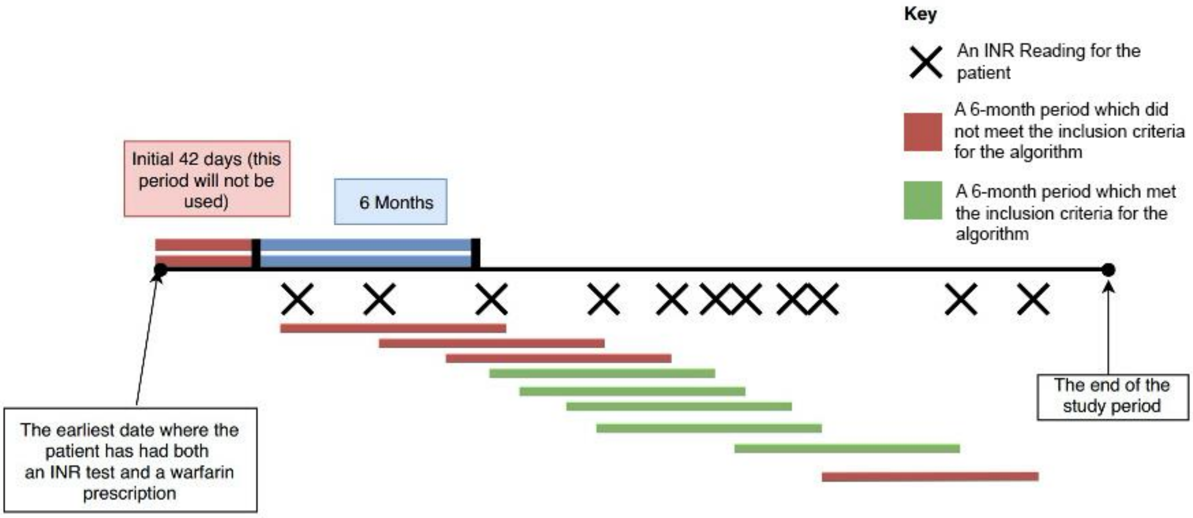

Supplementary Figure 1: TTR Temporal Window Identification Algorithm

The algorithm starts by identifying all of the INR tests the patient received during the study period. If there are 6-months of data present prior to the date of an INR test, the algorithm will identify a potential window to analyse the patient’s level of TTR control in the 6-month

window. The earliest point at which a window can begin is 42-days after the study start date for the patient. The algorithm will then perform a series of checks in order to check that the 6-month window meets the minimum requirements to calculate TTR, which are as follows:

- The patient must have had at least 4 INR readings in the window.
- There must be <u>a</u> gap of between 90 to 183-days from the first to the last reading within the 6-month period. This ensures we have a sufficient time period to calculate a TTR.
- There must be a gap of no more than 84-days between each reading and the subsequent reading in the sequence.

A window is excluded if it does not meet these minimum requirements. A window will always be exactly 6-months (183-days) long. If a patient does not have at least one valid window of data that we can analyse, they are excluded from our analysis. This will produce a series of rolling windows which we can use to analyse the patient’s level of control over time.

Examples of Exclusion Criteria

Below are a series of examples of valid and invalid temporal windows in the TTR temporal window algorithm.

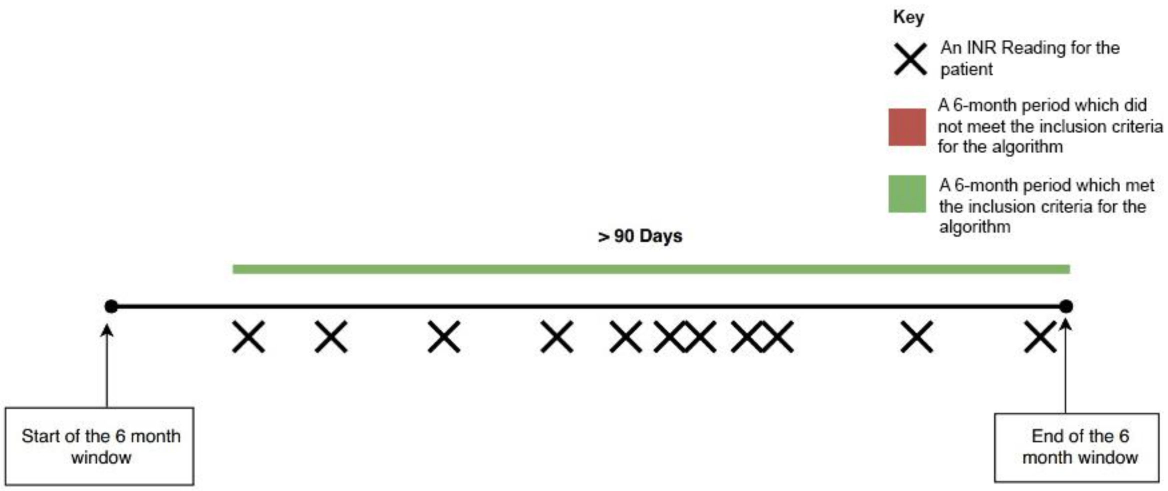

Supplementary Figure 2a: This is a valid window of analysis, because the patient has had more than 4 INR tests within the period, and there is a gap of more than 90-days from first to last reading:

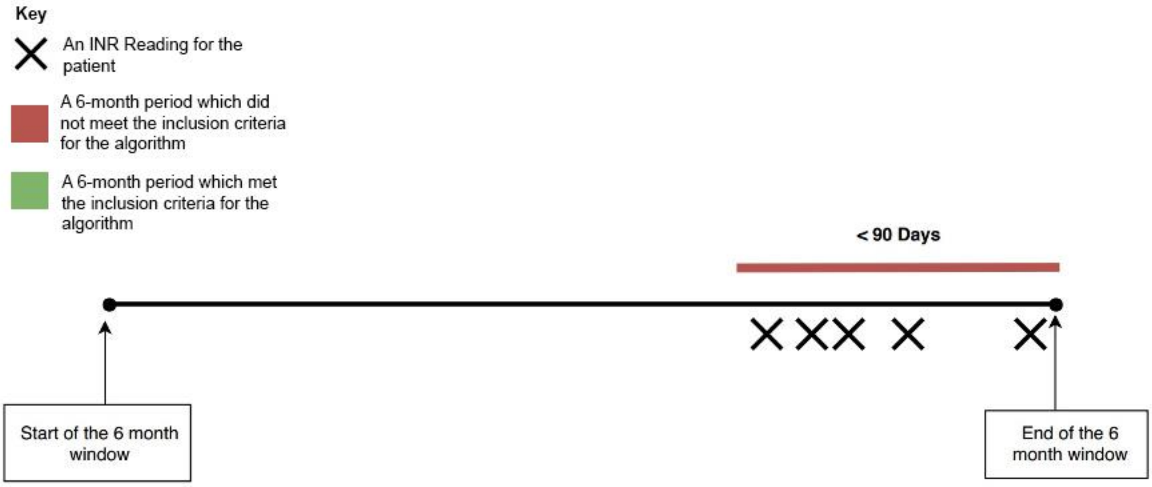

Supplementary Figure 2b: This window would be excluded because although there are more than 4 readings within the period, the gap between first and last reading is not sufficient to meet our minimum criteria:

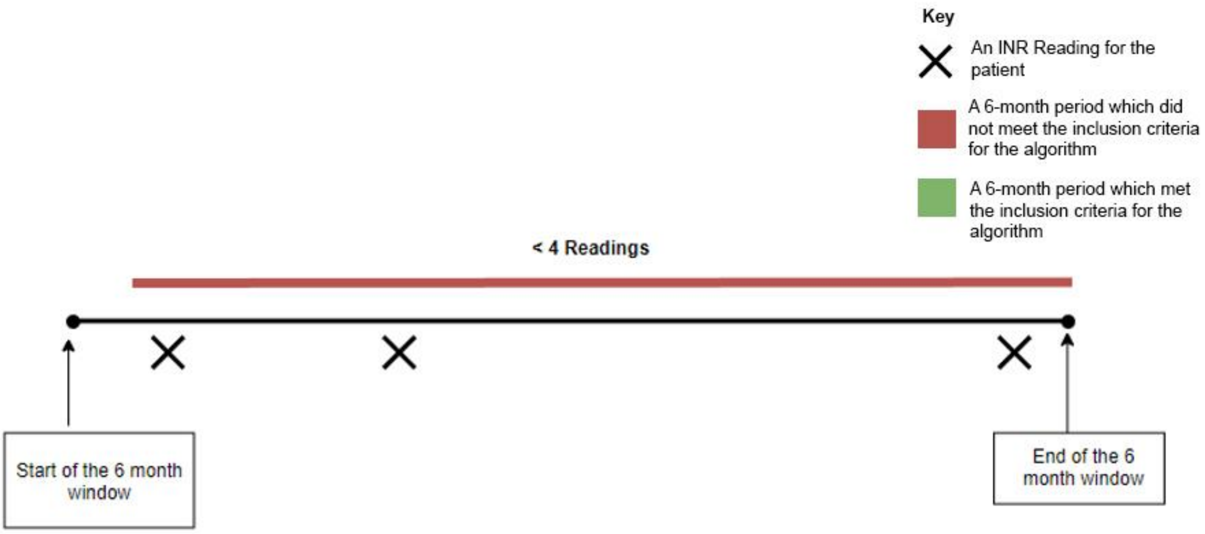

Supplementary Figure 2C: This window would be excluded because there are not 4 readings within the period.

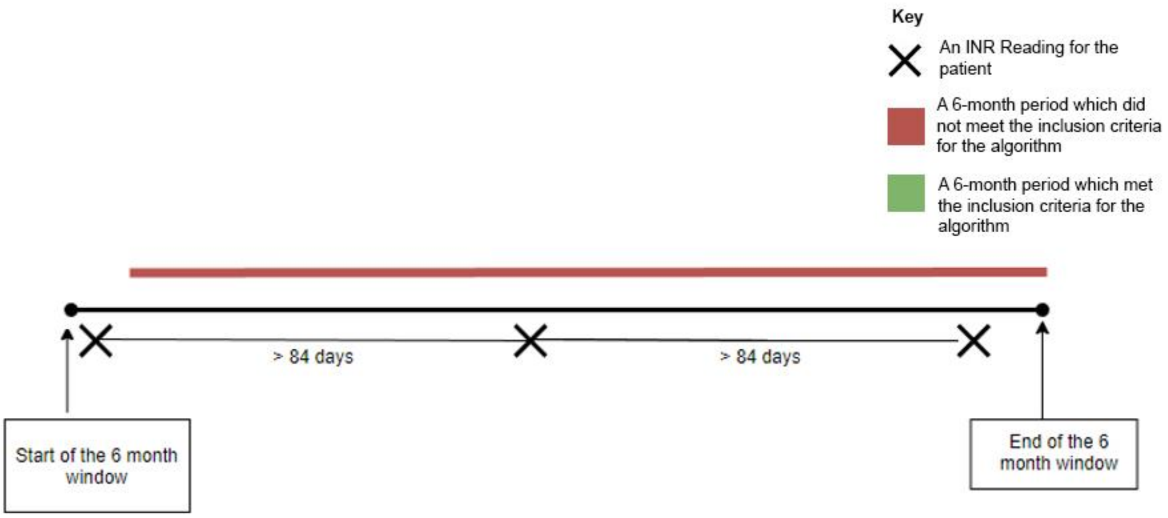

Supplementary Figure 2D: This period would be excluded because there are less than 4 readings, and the gap between each reading and the subsequent reading is greater than 84-days.

Supplementary Table 1a. Read codes for bleeding events for primary care data.

Bleeding event	Read Code	Description
Gastrointestinal	196B.	Painful rectal bleeding
Gastrointestinal	196C.	Painless rectal bleeding
Gastrointestinal	76191	Gastrot ligate bleed pt stom
Gastrointestinal	77341	Pt int sphincterot for haemorr
Gastrointestinal	77362	Manual reduct prolapse haemorr
Gastrointestinal	8512	Rectal packing-haemorr.control
Gastrointestinal	G850.	Oesophageal varices + bleeding
Gastrointestinal	G8520	Oesoph.varic.+dis.EC+bleeding
Gastrointestinal	J10y0	Haemorrhage of oesophagus
Gastrointestinal	J1101	Acute GU + haemorrhage
Gastrointestinal	J1111	Chronic GU + haemorrhage
Gastrointestinal	J11y1	Unspec. GU + haemorrhage
Gastrointestinal	J1201	Acute DU + haemorrhage
Gastrointestinal	J1211	Chronic DU + haemorrhage
Gastrointestinal	J12y1	Unspec. DU + haemorrhage
Gastrointestinal	J1301	Acute PU + haemorrhage
Gastrointestinal	J1311	Chronic PU + haemorrhage
Gastrointestinal	J13y1	Unspec. PU + haemorrhage
Gastrointestinal	J1401	Acute GJU + haemorrhage
Gastrointestinal	J1411	Chronic GJU + haemorrhage
Gastrointestinal	J14y1	Unspec. GJU + haemorrhage
Gastrointestinal	J1500	Acute haemorrhagic gastritis
Gastrointestinal	J5109	Bleeding diverticulosis
Gastrointestinal	J573.	Haemorrhage of rectum and anus
Gastrointestinal	J5730	Rectal haemorrhage
Gastrointestinal	J5731	Anal haemorrhage
Gastrointestinal	J573z	Haemorrhage of rectum/anus NOS
Gastrointestinal	J636.	Central haemorrhag necr liver
Gastrointestinal	J6702	Acute haemorrhag.pancreatitis
Gastrointestinal	J68..	Gastrointestinal haemorrhage
Gastrointestinal	J68z.	GIT haemorrhage unspecified
Gastrointestinal	J68z0	Gastric haemorrhage NOS
Gastrointestinal	J68z1	Intestinal haemorrhage NOS
Gastrointestinal	J68z2	Upper GI haemorrhage
Gastrointestinal	J68zz	GIT haemorrhage NOS
Gynecological	7F227	Pack to control P/N vag bleed
Gynecological	8513	Pack non-obst.uterine bleeding
Gynecological	K5311	Corpus luteum cyst haemorrhage
Gynecological	K55y3	Haemorrhage of cervix
Gynecological	K59y3	Intermenstrual bleeding
Gynecological	K5A1.	Postmenopausal bleeding
Gynecological	K5E..	Oth abnorm uterine vagin bleed
Gynecological	K5E0.	Abn uter bleed unrel menst cyc
Gynecological	K5E1.	Abnorm uterine bleeding, unsp
Gynecological	K5E2.	Abnor vagin bleed, unsp
Gynecological	K5Ez.	Abnor uterine vagin bleed unsp
Gynecological	Kyu9D	[X]O spc abnrm uterin+vg bleed
Respiratory	R0630	[D]Cough with haemorrhage
Respiratory	R0631	[D]Pulmonary haemorrhage NOS
Respiratory	Ryu02	[X]Haemor oth site resp passag
Respiratory	Ryu07	[X]Haemor from resp passag,uns

Supplementary Table 1a continued. Read codes for bleeding events for primary care data.

Bleeding event	Read Code	Description
Ocular	2BB5.	O/E - retinal haemorrhages
Ocular	2BB8.	O/E - vitreous haemorrhages
Ocular	F4045	Intra-ocular haemorrhage
Ocular	F4243	Ret.pigm.epith.haemorrh.detach
Ocular	F42y1	Superficial retinal haemorrh.
Ocular	F42y3	Deep retinal haemorrhage
Ocular	F42y4	Subretinal haemorrhage
Ocular	F42y5	Retinal haemorrhage NOS
Ocular	F436.	Choroidal haemorrhage/rupture
Ocular	F4360	Choroidal haemorrhage unspec.
Ocular	F4361	Expulsive choroidal haemorrh.
Ocular	F436z	Choroidal haemorr./rupture NOS
Ocular	F4372	Haemorrhagic choroidal detach.
Ocular	F4G32	Orbital haemorrhage
Ocular	F4H41	Optic nerve sheath haemorrhage
Ocular	F4K28	Vitreous haemorrhage
Ocular	F4K7.	Retrobulbar haemorrhage
Ocular	FyuH4	[X]Vitreous haemorrhage/dis CE
Urinary	K1381	Renal artery haemorrhage
Urinary	K13F.	Ureteric haemorrhage
Urinary	K167.	Haemorrhage into bladder wall
Urinary	K16y2	Bladder haemorrhage
Urinary	K19y4	Bleeding from urethra
Urinary	K221.	Prostatic congestion/haemorrh.
Urinary	K2211	Prostatic haemorrhage
Urinary	K221z	Prostatic congest/haemorrh NOS
Urinary	K2752	Corpus cavernosum haemorrhage
Urinary	K2861	Scrotal haemorrhage
Urinary	K2864	Testicular haemorrhage
Urinary	K286w	Male genital haemorrhage NOS
Miscellaneous	7404	Surg arrest bleeding int nose
Miscellaneous	7404y	Surg arrest bleed int nose OS
Miscellaneous	7404z	Surg arrest bleed int nose NOS
Miscellaneous	74213	Surg arr postop bleed adenoid
Miscellaneous	75175	Surg arr PO bleed tooth socket
Miscellaneous	75314	Surg arr PO bleed tonsil bed
Miscellaneous	77352	Inj sclerosing subst haemorrh
Miscellaneous	7H022	Reop chest arr PO bleed abd op
Miscellaneous	7H226	Reop abdo arrest post op bleed
Miscellaneous	7J013	Reopen cran arrest PO bleeding
Miscellaneous	7M0U4	Reexplor & arrest PO bleed NOC
Miscellaneous	851..	Haemorrhage control by packing
Miscellaneous	C063.	Thyroid haemorrhage/infarction
Miscellaneous	C0630	Thyroid haemorrhage
Miscellaneous	C063z	Thyroid haemorrh/infarct NOS
Miscellaneous	C12y1	Haemorrhage of parathyroid
Miscellaneous	C1542	Adrenal haemorrhage
Miscellaneous	D31X.	Haemorrhag condition, unsp
Miscellaneous	D31y.	Other haemorrhagic conditions
Miscellaneous	D31yz	Other haemorrhagic condit.NOS
Miscellaneous	D31z.	Haemorrhagic condition NOS

112

Supplementary Table 1a continued. Read codes for bleeding events for primary care data.		
**Bleeding event**	**Read Code**	**Description**
Miscellaneous	Dyu34	[X]Haemorrhag condition, unsp
Miscellaneous	J08zD	Angina bullosa haemorrhagica
Miscellaneous	R048.	[D]Throat haemorrhage
Miscellaneous	Ryu73	[X]Haemorrhage, NEC
Intracranial	662o.	Haemorrhagic stroke monitoring
Intracranial	G60..	Subarachnoid haemorrhage
Intracranial	G60..	Subarachnoid haemorrhage
Intracranial	G600.	Ruptured berry aneurysm
Intracranial	G601.	Subarac haem/carotd siph+bifur
Intracranial	G602.	Subarachd haem/mid cerebrl art
Intracranial	G603.	Subarachnd haem/ant commun art
Intracranial	G604.	Subarachn haem/post commun art
Intracranial	G605.	Subarachnd haem/basilar artery
Intracranial	G606.	Subarach haem/vertebral artery
Intracranial	G60X.	Subar haem,intracr art,unsp
Intracranial	G60z.	Subarachnoid haemorrhage NOS
Intracranial	G60z.	Subarachnoid haemorrhage NOS
Intracranial	G61..	Intracerebral haemorrhage
Intracranial	G61..	Intracerebral haemorrhage
Intracranial	G610.	Cortical haemorrhage
Intracranial	G610.	Cortical haemorrhage
Intracranial	G611.	Internal capsule haemorrhage
Intracranial	G611.	Internal capsule haemorrhage
Intracranial	G612.	Basal nucleus haemorrhage
Intracranial	G612.	Basal nucleus haemorrhage
Intracranial	G613.	Cerebellar haemorrhage
Intracranial	G613.	Cerebellar haemorrhage
Intracranial	G614.	Pontine haemorrhage
Intracranial	G614.	Pontine haemorrhage
Intracranial	G615.	Bulbar haemorrhage
Intracranial	G615.	Bulbar haemorrhage
Intracranial	G616.	External capsule haemorrhage
Intracranial	G616.	External capsule haemorrhage
Intracranial	G617.	Intrcereb haem,intraventriculr
Intracranial	G618.	Intracerebrl haem,multip local
Intracranial	G619.	Lobar cerebral haemorrhage
Intracranial	G619.	Lobar cerebral haemorrhage
Intracranial	G61X.	Intracer haem hemisph, unsp
Intracranial	G61X0	Left side intracereb haem unsp
Intracranial	G61X1	Right side intracer haem unsp
Intracranial	G61z.	Intracerebral haemorrhage NOS
Intracranial	G61z.	Intracerebral haemorrhage NOS
Intracranial	G62..	Oth/unspec intracranial bleed
Intracranial	G620.	Extradural haemorrh.-nontraum.

Supplementary Table 1a continued. Read codes for bleeding events for primary care data.

**Bleeding event**	**Read Code**	**Description**
Intracranial	G621.	Subdural haemorrhage-nontraum.
Intracranial	G623.	Subdural haemorrhage NOS
Intracranial	G62z.	Intracranial haemorrhage NOS
Intracranial	S620.	Cls trm subarach haemorrhage
Intracranial	S621.	Opn trm subarach haemorrhage
Intracranial	S622.	Cls trm subdural haemorrhage
Intracranial	S623.	Opn trm subdural haemorrhage
Intracranial	S624.	Cls trm extradural haemorrhage
Intracranial	S625.	Opn trm extradural haemorrhage
Intracranial	S626.	Epidural haemorrhage
Intracranial	S627.	Traum subarachnoid haemorrhage
Intracranial	S628.	Traumatic subdural haemorrhage

Supplementary Table 1b. *ICD-10* codes for bleeding events for secondary care data.

**Bleeding event**	**Read Code**	**Description**
Gastrointestinal	I850	Oesophageal varices with bleeding
Gastrointestinal	I983	Oesophageal varices with bleeding in diseases classified elsewhere
Gastrointestinal	K226	Gastro-oesophageal laceration-haemorrhage syndrome
Gastrointestinal	K290	Acute haemorrhagic gastritis
Gastrointestinal	K762	Central haemorrhagic necrosis of liver
Gastrointestinal	K922	Gastrointestinal haemorrhage, unspecified
Gastrointestinal	R041	Haemorrhage from throat
Gastrointestinal	R048	Haemorrhage from other sites in respiratory passages
Gastrointestinal	R049	Haemorrhage from respiratory passages, unspecified
Gastrointestinal	R58X	Haemorrhage, not elsewhere classified
Gastrointestinal	K921	Melaena
Gastrointestinal	K250	Gastric ulcer acute with haemorrhage
Gastrointestinal	K254	Gastric ulcer chronic or unspecified with haemorrhage
Gastrointestinal	K260	Duodenal ulcer acute with haemorrhage
Gastrointestinal	K264	Duodenal ulcer chronic or unspecified with haemorrhage
Gastrointestinal	K270	Peptic ulcer acute with haemorrhage
Gastrointestinal	K280	Gastro jejunal ulcer acute with haemorrhage
Gastrointestinal	K921	Melaena
Gastrointestinal	K922	Gastrointestinal haemorrhage unspecified
Gastrointestinal	K921	Melaena
Gastrointestinal	K922	Gastrointestinal haemorrhage unspecified
Gastrointestinal	K922	Gastrointestinal haemorrhage unspecified
Gynecological	N923	Ovulation bleeding
Gynecological	N924	Excessive bleeding in the premenopausal period
Gynecological	N93	Other abnormal uterine and vaginal bleeding
Gynecological	N930	Postcoital and contact bleeding
Gynecological	N938	Other specified abnormal uterine and vaginal bleeding
Gynecological	N939	Abnormal uterine and vaginal bleeding, unspecified
Gynecological	N950	Postmenopausal bleeding
Gynecological	N923	Ovulation bleeding

116

Supplementary Table 1b continued. *ICD-10* codes for bleeding events for secondary care data.

**Bleeding event**	**Bleeding event**	**Bleeding event**
Respiratory	R04	Haemorrhage from respiratory passages
Respiratory	J942	Haemothorax
Respiratory	K920	Haematemesis
Respiratory	R042	Haemoptysis
Respiratory	R048	Haemorrhage from other sites in respiratory passages
Ocular	H356	Retinal haemorrhage
Ocular	H313	Choroidal haemorrhage and rupture
Ocular	H356	Retinal haemorrhage
Ocular	H431	Vitreous haemorrhage
Ocular	H450	Vitreous haemorrhage in diseases classified elsewhere
Miscellaneous	D683	Haemorrhagic disorder due to circulating anticoagulants
Miscellaneous	D698	Other specified haemorrhagic conditions
Miscellaneous	D699	Haemorrhagic condition, unspecified
Miscellaneous	S064	Epidural haemorrhage
Miscellaneous	T792	Traumatic secondary and recurrent haemorrhage
Miscellaneous	T810	Haemorrhage and haematoma complicating a procedure, not elsewhere classified
Miscellaneous	Y60	Unintentional cut, puncture, perforation or haemorrhage during surgical and medical care
Urinary	N421	Congestion and haemorrhage of prostate
Urinary	N028	Recurrent and persistent haematuria other
Urinary	N029	Recurrent and persistent haematuria unspecified
Urinary	R31X	Unspecified haematuria
Intracranial	I60	Subarachnoid haemorrhage
Intracranial	I600	Subarachnoid haemorrhage from carotid siphon and bifurcation
Intracranial	I601	Subarachnoid haemorrhage from middle cerebral artery
Intracranial	I602	Subarachnoid haemorrhage from anterior communicating artery
Intracranial	I603	Subarachnoid haemorrhage from posterior communicating artery
Intracranial	I604	Subarachnoid haemorrhage from basilar artery
Intracranial	I605	Subarachnoid haemorrhage from vertebral artery
Intracranial	I606	Subarachnoid haemorrhage from other intracranial arteries

Supplementary Table 1b continued. ICD-10 codes for bleeding events for secondary care data.

Bleeding event	ICD 10 code	Bleeding event
Intracranial	I609	Subarachnoid haemorrhage, unspecified
Intracranial	I61	Intracerebral haemorrhage
Intracranial	I610	Intracerebral haemorrhage in hemisphere, subcortical
Intracranial	I611	Intracerebral haemorrhage in hemisphere, cortical
Intracranial	I612	Intracerebral haemorrhage in hemisphere, unspecified
Intracranial	I613	Intracerebral haemorrhage in brain stem
Intracranial	I614	Intracerebral haemorrhage in cerebellum
Intracranial	I615	Intracerebral haemorrhage, intraventricular
Intracranial	I616	Intracerebral haemorrhage, multiple localized
Intracranial	I618	Other intracerebral haemorrhage
Intracranial	I619	Intracerebral haemorrhage, unspecified
Intracranial	I62	Other nontraumatic intracranial haemorrhage
Intracranial	I620	Subdural haemorrhage (acute)(nontraumatic)
Intracranial	I621	Nontraumatic extradural haemorrhage
Intracranial	I629	Intracranial haemorrhage (nontraumatic), unspecified
Intracranial	S065	Traumatic subdural haemorrhage
Intracranial	S066	Traumatic subarachnoid haemorrhage

Supplementary Table 1c. Read codes for SSE events for primary care data

SSE event	Read Code	Description
Ischaemic stroke	G64z0	Brainstem infarction
Ischaemic stroke	G64z1	Wallenberg syndrome
Ischaemic stroke	G64z2	Left sided cerebral infarction
Ischaemic stroke	G64z3	Right sided cerebral infarct
Ischaemic stroke	G64z4	Infarction of basal ganglia
Ischaemic stroke	G661.	Anterior cerebral artery syn
Ischaemic stroke	G662.	Posterior cerebral artery syn
Ischaemic stroke	G663.	Brain stem stroke syndrome
Ischaemic stroke	G664.	Cerebellar stroke syndrome
Ischaemic stroke	G665.	Pure motor lacunar syndrome
Ischaemic stroke	G666.	Pure sensory lacunar syndrome
Ischaemic stroke	G63..	Precerebral arterial occlusion
Ischaemic stroke	G630.	Basilar artery occlusion
Ischaemic stroke	G631.	Carotid artery occlusion
Ischaemic stroke	G632.	Vertebral artery occlusion
Ischaemic stroke	G633.	Multip/bilat.precereb.art.occ.
Ischaemic stroke	G634.	Carotid artery stenosis
Ischaemic stroke	G63y.	Other precerebral artery occl.
Ischaemic stroke	G63y0	Cerebr infct/throm/precere art
Ischaemic stroke	G63y1	Cerebr infct/embol/precere art
Ischaemic stroke	G63z.	Precerebral artery occlus. NOS
Ischaemic stroke	G64..	Cerebral arterial occlusion
Ischaemic stroke	G640.	Cerebral thrombosis
Ischaemic stroke	G6400	Cerebr infct/throm/cerebrl art
Ischaemic stroke	G641.	Cerebral embolism
Ischaemic stroke	G6410	Cerebr infct/embol/cerebrl art
Ischaemic stroke	G64z.	Cerebral infarction NOS
Ischaemic stroke	G667.	Left sided CVA
Ischaemic stroke	G668.	Right sided CVA
Ischaemic stroke	G677.	Oc/st cere art,n rslt cer inft
Ischaemic stroke	G6770	Occlusn+stenos/midl cerebr art
Ischaemic stroke	G6771	Occlusn+stenos/anter cereb art
Ischaemic stroke	G6772	Occlusn+stenos/post cerebr art
Ischaemic stroke	G6773	Occlusn+stenos/cerebellar art
Ischaemic stroke	G6774	Occl/sten/mult+bilat cereb art
Ischaemic stroke	G683.	Sequelae/cerebral infarction
Ischaemic stroke	Gyu63	[X]Cereb in/uns oc,stn/cereb a
Ischaemic stroke	Gyu64	[X]Other cerebral infarction
Ischaemic stroke	Gyu65	[X]Oc+steno/o precerebral artr
Ischaemic stroke	Gyu66	[X]Oc+sten/o cerebral arteries
Ischaemic stroke	Gyu6C	[X]Seql/strok,n spc/h'm,infarc
Ischaemic stroke	Gyu6G	[X]Cer inf,un oc/st precer art

Supplementary Table 1c continued. Read codes for SSE events for primary care data.

SSE event	Read Code	Description
Haemorrhagic stroke	G61..	Intracerebral haemorrhage
Haemorrhagic stroke	G610.	Cortical haemorrhage
Haemorrhagic stroke	G611.	Internal capsule haemorrhage
Haemorrhagic stroke	G612.	Basal nucleus haemorrhage
Haemorrhagic stroke	G613.	Cerebellar haemorrhage
Haemorrhagic stroke	G614.	Pontine haemorrhage
Haemorrhagic stroke	G615.	Bulbar haemorrhage
Haemorrhagic stroke	G616.	External capsule haemorrhage
Haemorrhagic stroke	G617.	Intrcereb haem,intraventriculr
Haemorrhagic stroke	G618.	Intracerebrl haem,multip local
Haemorrhagic stroke	G61z.	Intracerebral haemorrhage NOS
Haemorrhagic stroke	G62z.	Intracranial haemorrhage NOS
Haemorrhagic stroke	G681.	Seq/intracerebral haemorrhage
Haemorrhagic stroke	G682.	Seq/oth nontraum intrcran haem
Haemorrhagic stroke	Gyu62	[X]Oth intracerebrl h'morrhage
Haemorrhagic stroke	Gyu6F	[X]Intracer haem hemisph, unsp
Systemic embolus	D4157	Splenic infarction
Systemic embolus	G74..	Arterial embolism/thrombosis
Systemic embolus	G740.	Embolus/thrombus abdom.aorta
Systemic embolus	G741.	Embolus/thromb.thoracic aorta
Systemic embolus	G742.	Embolus/thromb.arm/leg artery
Systemic embolus	G7420	Embolus/thrombus brachial art.
Systemic embolus	G7421	Embolus/thrombus radial artery
Systemic embolus	G7422	Embolus/thrombus ulnar artery
Systemic embolus	G7423	Embolus/thromb.arm artery NOS
Systemic embolus	G7424	Embolus/thrombus femoral art.
Systemic embolus	G7425	Embolus/thromb.popliteal art.
Systemic embolus	G7426	Embolus/thromb.ant.tibial art.
Systemic embolus	G7427	Embolus/thromb.dors.pedis art.
Systemic embolus	G7428	Embolus/thromb.post.tibial art
Systemic embolus	G7429	Embolus/thromb.leg artery NOS
Systemic embolus	G742z	Periph.arterial embolism NOS
Systemic embolus	G74y.	Embolus/thrombus other artery
Systemic embolus	G74y0	Embolus/thromb.com.iliac art.
Systemic embolus	G74y1	Embolus/thromb.int.iliac art.
Systemic embolus	G74y2	Embolus/thromb.ext.iliac art.
Systemic embolus	G74y3	Embolus/thromb iliac art.unsp.
Systemic embolus	G74y5	Embolus/throm.subclavian art.
Systemic embolus	G74y6	Embolus/thromb.splenic artery
Systemic embolus	G74y7	Embolus/thromb.axillary artery
Systemic embolus	G74y8	Embolus/thromb.coeliac artery
Systemic embolus	G74y9	Embolus/thromb.hepatic artery
Systemic embolus	G74yz	Embolus/thrombus other art.NOS
Systemic embolus	G74z.	Arterial embolus/thromb.NOS
Systemic embolus	Gyu75	[X]Embolsm+thromb/oth arteries

Supplementary Table 1d. *ICD-10* codes for SEE events for secondary care data		
**SSE event**	**ICD-10 code**	**Description**
Ischaemic stroke	I630	Cerebral infarct due to thrombosis of precerebral arteries
Ischaemic stroke	I631	Cerebral infarction due to embolism of precerebral arteries
Ischaemic stroke	I632	Cereb infarct due unsp occlusion or stenos precerebrl arts
Ischaemic stroke	I633	Cerebral infarction due to thrombosis of cerebral arteries
Ischaemic stroke	I634	Cerebral infarction due to embolism of cerebral arteries
Ischaemic stroke	I635	Cerebrl infarct due unspec occlusion or stenos cerebrl arts
Ischaemic stroke	I636	Cereb infarct due cerebral venous thrombosisnonpyogenic
Ischaemic stroke	I638	Other cerebral infarction
Ischaemic stroke	I639	Cerebral infarctionunspecified
Ischaemic stroke	I650	Occlusion and stenosis of vertebral artery
Ischaemic stroke	I651	Occlusion and stenosis of basilar artery
Ischaemic stroke	I652	Occlusion and stenosis of carotid artery
Ischaemic stroke	I653	Occlusion and stenosis of multip and bilat precerebrl arts
Ischaemic stroke	I658	Occlusion and stenosis of other precerebral artery
Ischaemic stroke	I659	Occlusion and stenosis of unspecified precerebral artery
Ischaemic stroke	I660	Occlusion and stenosis of middle cerebral artery
Ischaemic stroke	I661	Occlusion and stenosis of anterior cerebral artery
Ischaemic stroke	I662	Occlusion and stenosis of posterior cerebral artery
Ischaemic stroke	I663	Occlusion and stenosis of cerebellar arteries
Ischaemic stroke	I664	Occlusion and stenosis of multiple and bilat cerebrl arts
Ischaemic stroke	I668	Occlusion and stenosis of other cerebral artery
Ischaemic stroke	I669	Occlusion and stenosis of unspecified cerebral artery
Ischaemic stroke	G458	Other transient cerebral ischaemic attacks and related synd
Ischaemic stroke	I630	Cerebral infarct due to thrombosis of precerebral arteries
Ischaemic stroke	I631	Cerebral infarction due to embolism of precerebral arteries
Ischaemic stroke	I632	Cereb infarct due unsp occlusion or stenos precerebrl arts
Ischaemic stroke	I633	Cerebral infarction due to thrombosis of cerebral arteries

125

Supplementary Table 1d continued. *ICD-10* codes for SEE events for secondary care data

SSE event	ICD-10 code	Description
Ischaemic stroke	I664	Occlusion and stenosis of multiple and bilat cerebrl arts
Ischaemic stroke	I668	Occlusion and stenosis of other cerebral artery
Ischaemic stroke	I669	Occlusion and stenosis of unspecified cerebral artery
Ischaemic stroke	G458	Other transient cerebral ischaemic attacks and related synd
Systemic embolism	I740	Embolism and thrombosis of abdominal aorta
Systemic embolism	I741	Embolism and thrombosis of other and unspec parts of aorta
Systemic embolism	I742	Embolism and thrombosis of arteries of upper extremities
Systemic embolism	I743	Embolism and thrombosis of arteries of lower extremities
Systemic embolism	I744	Embolism and thrombosis of arteries of extremitiesunspec
Systemic embolism	I745	Embolism and thrombosis of iliac artery
Systemic embolism	I748	Embolism and thrombosis of other arteries
Systemic embolism	I749	Embolism and thrombosis of unspecified artery
Haemorrhagic stroke	I610	Intracerebral haemorrhage in hemispheresubcortical
Haemorrhagic stroke	I611	Intracerebral haemorrhage in hemispherecortical
Haemorrhagic stroke	I612	Intracerebral haemorrhage in hemisphereunspecified
Haemorrhagic stroke	I613	Intracerebral haemorrhage in brain stem
Haemorrhagic stroke	I614	Intracerebral haemorrhage in cerebellum
Haemorrhagic stroke	I615	Intracerebral haemorrhageintraventricular
Haemorrhagic stroke	I616	Intracerebral haemorrhagemultiple localized
Haemorrhagic stroke	I618	Other intracerebral haemorrhage
Haemorrhagic stroke	I619	Intracerebral haemorrhageunspecified
Haemorrhagic stroke	I629	Intracranial haemorrhage (nontraumatic)unspecified

Table 2. Cohort baseline characteristics

	**Overall cohort**<br>*n* (%)
	Cohort<br>N (%)
	N= 37,638
Age	
18-64	6,089 1(16·2)
65-74	12,508 (33·2)
75+	19,041 (50·6)
Female	16,082 (42·7)
Deprivation index* (quintile)	
1 (most deprived)	6,184 (17·1)
2	7,083 (19·6)
3	8,057 (22·3)
4	7,070 (19·5)
5 (least deprived)	7,780 (21·5)
CHA_2DS_2-VASc score	
0 and 1	4,233 (11·2)
2	6,513 (17·3)
3	8,493 (22·6)
4	8,256 (21·9)
5	5,351 (14·2)
6	3,157 (8·4)
≥7	1,635 (4·3)
Heart failure	8,840 (23·5)
Hypertension	24,753 (65·8)
Diabetes	8,049 (21·4)
Ischemic stroke	7,811 (20·8)
Thromboembolism	528 (1·4)
Ischemic heart disease	11,480 (30·5)
Peripheral Vascular Disease	2,314 (6·1)
Liver disease	729 (1·9)
Chronic Kidney disease (stage 4+)	459 (1·2)
Excessive alcohol intake	998 (2·7)
Any prior bleeding	5,208 (13·8)

* Deprivation index used is the WIMD quintile.

Supplementary Table 3. Multivariable Cox-regression model for hazard of stroke and systemic embolism and bleeding events determined by poor INR control according to ESC/US criteria.

Results are adjusted for CHA_2DS_2-VASc score and deprivation quintiles. Any changes in INR control status for individuals over time were included in the model as a time dependent variable.

	**Stroke and systemic embolism** HR (95%CI), *P* value	**Bleeding** HR (95%CI), *P* value
Poor INR control	1·69 (1·54-1·86), <0·001	1·43 (1·35-1·51), <0·001
CHA_2DS_2-VASc score		
0 &1	Reference	Reference
2	1·96 (1·50-2·57), <0·001	1·22 (1·09-1·37), <0·001
3	2·93 (2·28-3·77), <0·001	1·45 (1·29-1·61), <0·001
4	3·76 (2·93-4·82), <0·001	1·54 (1·38-1·72), <0·001
5	5·47 (4·26-7·03), <0·001	1·66 (1·48-1·87), <0·001
6	6·79 (5·22-8·81), <0·001	1·68 (1·47-1·92), <0·001
7	9·08 (6·85-12·06), <0·001	1·98 (1·69-2·33), <0·001
Deprivation index (quintiles)*		
1 (most deprived)	Reference	Reference
2	0·89 (0·76-1·04), 0·13	0·99 (0·91-1·10), 0·99
3	0·88 (0·76-1·03), 0·12	0·96 (0·87-1·05), 0·36
4	0·99 (0·85-1·15), 0·89	0·96 (0·88-1·06), 0·45
5 (least deprived)	0·92 (0·79-1·07), 0·26	1·00 (0·92-1·09), 0·97

*Deprivation index was calculated using the Welsh Index of Multiple Deprivation 2011 quintiles.

Supplementary Table 4. Multivariable Cox-regression model for hazard of stroke and systemic embolism and bleeding events determined by poor INR control ESC/US.

Results are adjusted for individual components of CHA_2DS_2-VASc score, plus baseline characteristics. Any changes in INR control status for individuals over time were included in the model as a time dependent variable.

	**Stroke and systemic embolism** HR (95%CI), *P* value	**Bleed** HR (95%CI), *P* value
Poor INR control	1·73 (1·58-1·90), <0·001	1·42 (1·34-1·50), <0·001
Female	1·11 (1·01-1·22, 0·03	0·85 (0·80-0·90), <0·001
Age		
<65	Reference	Reference
65-74	1·71 (1·4302·03), <0·001	1·27 (1·16-1·38), <0·001
≥75	2·80 (2·36-3·32), <0·001	1·62 (1·49-1·77), <0·001
Excessive alcohol consumption	1·32 (0·97-1·80), 0·07	0·96 (0·80-1·16), 0·69
Prior bleeding events	1·15 (1·01-1·32), 0·03	1·55 (1·44-1·67), <0·001
Hypertension	1·26 (1·14-1·41), <0·001	1·05 (0·99-1·12), 0·07
Liver disease	0·99 (0·69-1·44), 0·99	1·15 (0·93-1·41), 0·19
Diabetes	1·20 (1·07-1·34), 0·001	1·12 (1·05-1·20), <0·001
Heart failure	1·03 (0·93-1·16), 0·55	1·08 (1·01-1·15), 0·03
Ischemic heart disease	0·99 (0·90-1·10), 0·93	1·16 (1·10-1·23), <0·001
Ischemic stroke	2·17 (1·97-2·39), <0·001	1·10 (1·03-1·17), 0·006
PVD*	1·79 (1·54-2·10), <0·001	1·11 (0·98-1·24), 0·09
Thromboembolism	1·25 (0·89-1·74), 0·20	0·93 (0·73-1·20), 0·59
CKD (stage 4+)	1·20 (0·79-1·83), 0·40	1·44 (1·13-1·85), 0·003

*PVD indicates Peripheral vascular Disease; CKD indicates Chronic Kidney disease.

Supplementary Table 5. Stroke and systemic embolism event rate according to INR guideline criteria.

Guideline criteria	INR control	Number of SSE	Number of patient years	Event rate per 100 patient years
NICE				
	Adequate	947	92,999	1·0
	Poor	890	42,682	1·8
	N/A*	485	2,386	20·3
ESC/US				
	Adequate	814	83,315	0·9
	Poor	1,023	58,365	1·8
	N/A*	485	2,386	20·3

*NA indicates bleeds occurring during periods where it was not possible to calculate INR control.

Supplementary Table 6. Bleeding event rate according to INR guideline criteria.				
Guideline criteria	INR control	Number of bleeds	Number of patient years	Event rate per 100 patient years
NICE				
	Adequate	2,926	85,445	3·4
	Poor	2,113	44,273	4·8
	N/A*	1,265	2,965	42·7
ESC/US				
	Adequate	2,538	76,580	3·3
	Poor	2,501	53,138	4·7
	N/A*	1,265	2,965	42·7
*NA indicates bleeds occurring during periods where it was not possible to calculate INR control.				

Supplementary Table 7a Comparisons between those with deprivation index present and missing from the final cohort included in the SSE analysis. N= 35,891.			
	Deprivation index present	Deprivation index missing	*P* value
	N=34,495	N=1,396	
Mean age	73·6 (SD=9·6)	74·2 (SD=10·2)	0·03
CHA_2DS_2-VASc (mean)	3·5 (SD=1·7)	3·5 (SD=1·7)	0·86
	N(%)	N(%)	
Female	14,573(42·8)	575 (41·2)	0·24
Heart failure	8,106 (23·5)	369 (26·4)	0·01
Hypertension	22,710 (65·8)	871 (62·4)	0·08
Diabetes	7,388 (21·4)	278 (19·9)	0·18
Ischaemic stroke	6,794 (19·7)	274 (19·6)	0·95
Ischaemic heart disease	10,487 (30·4)	422 (30·2)	0·89
Thromboembolism	456 (1·3)	22 (1·6)	0·42
PVD	2,104 (6·1)	82 (5·9)	0·73
Liver disease	659 (1·9)	28 (2·0)	0·80
Chronic kidney disease (stage 4+)	416 (1·2)	19 (1·4)	0·60
Excessive alcohol intake	898 (2·6)	40 (2·9)	0·55
Any prior bleeding	4,748 (13·8)	171 (12·2)	0·11

Supplementary Table 7b Comparisons between those with deprivation index present and missing from the final cohort included in the bleeding analysis. N= 35,035.

	Deprivation index present	Deprivation index missing	P value
	N=33,662	N=1,373	
	Mean (SD)	Mean (SD)	
Mean age	73·6 (9·6)	74·3 (10·2)	0·03
CHA_2DS_2-VASc (mean)	3·5 (1·7)	3·5 (1·8)	0·71
	N (%)	N(%), p	
Female	14,473 (43·0)	568 (41·4)	0·23
Heart failure	7,918 (23·5)	365 (26·6)	0·009
Hypertension	22,191 (65·9)	858 (62·5)	0·009
Diabetes	7,195 (21·4)	281 (20·5)	0·42
Ischaemic stroke	6,999 (20·8)	291 (21·3)	0·67
Ischaemic heart disease	10,190 (30·3)	418 (30·4)	0·89
Thromboembolism	463 (1·4)	23 (1·7)	0·35
PVD	2,058 (6·1)	81 (5·9)	0·75
Liver disease	640 (1·9)	30 (2·2)	0·45
Chronic kidney disease (stage 4+)	398 (1·2)	19 (1·4)	0·50
Excessive alcohol intake	891 (2·6)	44 (3·2)	0·21
Any prior bleeding	4,490 (13·3)	167 (12·2)	0·21

Supplementary Table 8a. Cohort characteristics and comparisons to patients with inadequate number of INR readings to calculate INR control prior to a SSE and those with inadequate number of INR results to calculate INR control across any period during the study.

	Cohort analysed	Insufficient number of INR results prior to SSE	Inadequate number of INR results to calculate INR control at any point
	N=35,891	N=1,747	N=18,379
Mean age at entry into study	73·6 (9·7)	74·4 (9·5), P<0·001	*
Mean age of diagnosis of AF	70·6 (10·6)	71·7 (10·2), P<0·001	70·8 (11·7), P<0·001
Age category at entry into study		P=0·006	*
18-64	5,853 (16·3)	236 (13·5)	*
65-74	11,925 (33·2)	583 (33·4)	*
75+	18,113 (50·5)	928 (53·1)	*
Female	15,328 (42·7)	754 (43·2), 0·71	7858 (42·8), P=0·92
Deprivation quintile		P<0·001	P<0·001
1 (most deprived)	5,839 (16·9)	345 (20·5)	2,180 (18·5)
2	6,728 (19·5)	6,728 (21·1)	2,195 (18·7)
3	7,690 (22·3)	7,690 (22·3)	2,570 (21·8)
4	6,770 (19·7)	291 (17·3)	2,187 (18·6)
5 (least deprived)	7,459 (21·6)	321 (19·1)	2,636 (22·4)
CHA_2DS_2-VASc (mean)	3·5 (1·7)	4·04 (1·7), P<0·001	3·7 (1·8), P<0·001
CHA_2DS_2-VASc		P<0·001	P<0·001
0 &1	4,122 (11·5)	111 (6·4)	1,477 (12·1)
2	6,272 (17·5)	241 (13·8)	1,702 (13·9)
3	8,154 (22·7)	339 (19·4)	2,487 (20·3)
4	7,901 (22·0)	355 (20·3)	2,632 (21·5)
5	5,026 (14·0)	325 (18·6)	1,907 (15·6)
6	2,914 (8·1)	243 (13·9)	1,234 (10·1)
≥7	1,502 (4·2)	133 (7·6)	808 (6·6)
Heart failure	8,475 (23·6)	365 (20·9), P=0·009	6,366 (34·6), P<0·001
Hypertension	23,581 (65·7)	1,172 (67·1), P=0·23	11,590 (63·1), P<0·001
Diabetes	7,666 (21·4)	383 (21·9), P=0·57	4,643 (25·3), P<0·001
Ischaemic stroke	7,068 (19·7)	743 (42·5), P<0·001	4,220 (23·0), P<0·001
Ischaemic heart disease	10,909 (30·4)	571 (32·7), P=0·04	6,627 (36·1), P<0·001
Thromboembolism	478 (1·3)	50 (2·9), <0·001	334 (1·8), P<0·001
PVD	2,186 (6·1)	128 (7·3), P=0·03	1,584 (8·6), P<0·001
Liver disease	687 (1·9)	42 (2·4), P=0·15	727 (4·0), P<0·001
Chronic kidney disease (stage 4+)	435 (1·2)	24 (1·4), P=0·55	551 (3·0), P<0·001
Excessive alcohol intake	938 (2·6)	60 (3·4), P=0·04	627 (3·4), P<0·001
Any prior bleeding	4,919 (13·7)	289 (16·5), P=0·001	4,188 (22·8), P<0.001

*Information not entered as this group were not included in the analysis.

Supplementary Table 8b. Cohort characteristics and comparisons to patients with inadequate number of INR readings to calculate INR control prior to a bleed and those with inadequate number of INR results to calculate INR control across any period during the study.

	Cohort analysed	Inadequate number of INR results prior to bleed	Inadequate number of INR results to calculate INR control at any point
	N= 35,035	N=2,603	N=18,379
Mean age entry into study	73·4 (SD=9·4)	73·7 (SD=9·6), P=0·21	*
Mean age of diagnosis of AF	70·6 (SD=10·6)	71.7 (SD=10.2), P<0.001	70·8 (SD=11·7), P=0·09
	N(%)	N(%)	N(%)
Age category at entry into study		P=0·36	*
18-64	5,679 (16·2)	410 (15·8)	*
65-74	11,610 (33·1)	898 (34·5)	*
75+	17,746 (50·7)	1,295 (49·8)	*
Female	15,041 (42·9)	1,041 (40·0), P=0·003	7,858 (42·8), P=0·70
Deprivation quintile		P=0·005	P<0·001
1 (most deprived)	5,732 (17·0)	452 (18·0)	2,180 (18·5)
2	6,573 (19·5)	510 (20·3)	2,195 (18·7)
3	7,450 (22·1)	607 (24·2)	2,570 (21·8)
4	6,611 (19·6)	459 (18·3)	2,187 (18·6)
5 (least deprived)	7,296 (21·7)	484 (19·3)	2,636 (22·4)
CHA_2DS_2-VASc (mean)	3·5 (1·6)	3·5 (1·7), P=0·10	3·7 (SD= 1·8) P<0·001
CHA_2DS_2-VASc		P= 0·008	P <0·001
0 &1	3,971 (11·3)	262 (10·1)	1,477 (12·1)
2	6,018 (17·2)	495 (19·0)	1,702 (13·9)
3	7,859 (22·4)	634 (24·4)	2,487 (20·3)
4	7,692 (22·0)	564 (21·7)	2,632 (21·5)
5	4,999 (14·3)	352 (13·5)	1,907 (15·6)
≥6	2,962 (8·5)	195 (7·5)	1,234 (10·1)
7	1,534 (4·4)	101 (3·9)	808 (6·6)
Heart failure	8,283 (23·6)	557 (21·4), P= 0·009	6,366 (34·6), P=<0·001
Hypertension	23,049 (65·8)	1704 (65·5), P= 0·74	11,590 (63·1), P<0.001
Diabetes	7,476 (21·3)	573 (22·0), P=0·42	4,643 (25·3), P<0·001
Ischaemic stroke	7,291 (20·8)	520 (20·0), P=0·31	4,220 (23·0), P<0·001
Ischaemic heart disease	486 (1·4)	872 (33·5), P=0·001	6,627 (36·1), P<0·001
Thromboembolism	10,608 (30·3)	42 (1·6), P=0·34	334 (1·8), P<0·001
PVD	2,139 (6·1)	175 (6·7), P=0·21	1,584 (8·6), P<0·001
Liver disease	670 (1·9)	59 (2·3), P=0·20	727 (4·0), P<0·001
Chronic kidney disease (stage 4+)	417 (1·2)	42 (1·6), P=0·06	551 (3·0), P<0·001
Excessive alcohol intake	935 (2·7)	63 (2·4), P=0·45	627 (3·4), P<0·001
Any prior bleeding	4,657 (13·3)	551 (21·2), P<0·001	4,188 (22·8), P<0·001

*Information not entered as this group were not included in the analysis.
