# Supplementary material for: SAIL study of stroke, systemic embolism and bleeding outcomes with warfarin anticoagulation in non-valvular atrial fibrillation (S4-BOW-AF)
Source: Eur Heart J Open. 2023 Apr 13;3(3):oead037. doi: 10.1093/ehjopen/oead037 (PMC10153743; doi:10.1093/ehjopen/oead037)
Supplement: oead037_Supplementary_Data [file oead037_supplementary_data.zip › S4_strobe.doc]

STROBE Statement—checklist of items that should be included in reports of observational studies

|  | Item No | Recommendation |
| --- | --- | --- |
| **Title and abstract** | 1 | (*a*) Indicate the study’s design with a commonly used term in the title or the abstract  ‘Individual-level population-scale linked patient data’ is included in the abstract (pg 2) and detailed in the methods section pg 5. |
|  |  | (*b*) Provide in the abstract an informative and balanced summary of what was done and what was found  This is provided on pg 2 |
| Introduction | | |
| Background/rationale | 2 | Explain the scientific background and rationale for the investigation being reported  Provided on pg 4 |
| Objectives | 3 | State specific objectives, including any prespecified hypotheses  Aims of the study are provided on pg 5 |
| Methods | | |
| Study design | 4 | Present key elements of study design early in the paper  This is detailed at the start of the method on pg5 |
| Setting | 5 | Describe the setting, locations, and relevant dates, including periods of recruitment, exposure, follow-up, and data collection  Cohort selection and observation period is presented on pg 5. An explanation of the data sources is provided in the supplement. |
| Participants | 6 | (*a*) *Cohort study*—Give the eligibility criteria, and the sources and methods of selection of participants. Describe methods of follow-up  Cohort selection is provided on pg5  *Case-control study*—Give the eligibility criteria, and the sources and methods of case ascertainment and control selection. Give the rationale for the choice of cases and controls  *Cross-sectional study*—Give the eligibility criteria, and the sources and methods of selection of participants |
|  |  | (*b*) *Cohort study*—For matched studies, give matching criteria and number of exposed and unexposed  *Case-control study*—For matched studies, give matching criteria and the number of controls per case |
| Variables | 7 | Clearly define all outcomes, exposures, predictors, potential confounders, and effect modifiers. Give diagnostic criteria, if applicable  Adverse events of interest is provided on page 7, diagnostic codes of interest are supplied in the supplementary tables 1a-1d. Our models accounted for potential cofounders describe on page 9 and in the results tables. |
| Data sources/ measurement | 8* | For each variable of interest, give sources of data and details of methods of assessment (measurement). Describe comparability of assessment methods if there is more than one group  Calculation of INR control and identification of adverse events is detailed on pg 6. Data sources are described in the supplement. |
| Bias | 9 | Describe any efforts to address potential sources of bias  Detailed inclusion/exclusion criteria limited entry of patients prescribed warfarin for other indications pg 5&6  Assessment of missing data provided on pg 9 and supplementary tables (suppl pg 23-27) |
| Study size | 10 | Explain how the study size was arrived at  Consort figure provided on pg32 |
| Quantitative variables | 11 | Explain how quantitative variables were handled in the analyses. If applicable, describe which groupings were chosen and why |
| Statistical methods | 12 | (a) Describe all statistical methods, including those used to control for confounding.   Statistical methods are described on pg 8. |
|  |  | (*b*) Describe any methods used to examine subgroups and interactions |
|  |  | (b) Explain how missing data were addressed   Comparisons are made for those with deprivation index present and missing (supplementary tables 71 and 7b). Cohort characteristics of those with inadequate numbers of INR results prior to bleed or SSE are presented on supplementary tables 8a and 8b. |
|  |  | (*d*) *Cohort study*—If applicable, explain how loss to follow-up was addressed  *Case-control study*—If applicable, explain how matching of cases and controls was addressed  *Cross-sectional study*—If applicable, describe analytical methods taking account of sampling strategy |
|  |  | (*e*) Describe any sensitivity analyses |

Continued on next page

| Results | | |
| --- | --- | --- |
| Participants | 13* | (a) Report numbers of individuals at each stage of study—eg numbers potentially eligible, examined for eligibility, confirmed eligible, included in the study, completing follow-up, and analysed. This is presented in the consort figure 1. |
|  |  | (b) Give reasons for non-participation at each stage |
|  |  | (c) Consider use of a flow diagram This is presented in the consort figure 1. |
| Descriptive data | 14* | (a) Give characteristics of study participants (eg demographic, clinical, social) and information on exposures and potential confounders Presented in tables 1 and 4. |
|  |  | (b) Indicate number of participants with missing data for each variable of interest. Missing deprivation index is presented in table 1 and 4 |
|  |  | (c) *Cohort study*—Summarise follow-up time (eg, average and total amount) Summarised in the text on page 10 |
| Outcome data | 15* | *Cohort study*—Report numbers of outcome events or summary measures over time  Event rates are presented on pages 12 and 13 and supplementary table 5 and 6. |
|  |  | *Case-control study—*Report numbers in each exposure category, or summary measures of exposure |
|  |  | *Cross-sectional study—*Report numbers of outcome events or summary measures |
| Main results | 16 | (a) Give unadjusted estimates and, if applicable, confounder-adjusted estimates and their precision (eg, 95% confidence interval). Make clear which confounders were adjusted for and why they were included   Univariable hazard ratios are presented on pages 11 and 13. Multivariable models are presented in tables 2, 3, 5 and 6 |
|  |  | (*b*) Report category boundaries when continuous variables were categorized |
|  |  | (*c*) If relevant, consider translating estimates of relative risk into absolute risk for a meaningful time period. |
| Other analyses | 17 | Report other analyses done—eg analyses of subgroups and interactions, and sensitivity analyses |
| Discussion | | |
| Key results | 18 | Summarise key results with reference to study objectives Presented in the discussion page 14 |
| Limitations | 19 | Discuss limitations of the study, taking into account sources of potential bias or imprecision. Discuss both direction and magnitude of any potential bias.  Limitations are presented on page 17. |
| Interpretation | 20 | Give a cautious overall interpretation of results considering objectives, limitations, multiplicity of analyses, results from similar studies, and other relevant evidence  Provided on page 15 |
| Generalisability | 21 | Discuss the generalisability (external validity) of the study results  Discusses at the top of page 16 |
| Other information | | |
| Funding | 22 | Give the source of funding and the role of the funders for the present study and, if applicable, for the original study on which the present article is based |

A funding statement is provided on page 21

*Give information separately for cases and controls in case-control studies and, if applicable, for exposed and unexposed groups in cohort and cross-sectional studies.

**Note:** An Explanation and Elaboration article discusses each checklist item and gives methodological background and published examples of transparent reporting. The STROBE checklist is best used in conjunction with this article (freely available on the Web sites of PLoS Medicine at http://www.plosmedicine.org/, Annals of Internal Medicine at http://www.annals.org/, and Epidemiology at http://www.epidem.com/). Information on the STROBE Initiative is available at www.strobe-statement.org.
